# Esophageal extracellular matrix hydrogel mitigates metaplastic change in a dog model of Barrett’s esophagus

**DOI:** 10.1126/sciadv.aba4526

**Published:** 2020-07-01

**Authors:** Juan Diego Naranjo, Lindsey T. Saldin, Eric Sobieski, Lina M. Quijano, Ryan C. Hill, Patrick G. Chan, Crisanto Torres, Jenna L. Dziki, Madeline C. Cramer, Yoojin C. Lee, Rohit Das, Anant K. Bajwa, Rania Nossair, Molly Klimak, Lucile Marchal, Shil Patel, Sachin S. Velankar, Kirk C. Hansen, Kevin McGrath, Stephen F. Badylak

**Affiliations:** 1 McGowan Institute for Regenerative Medicine, University of Pittsburgh, Pittsburgh, PA 15219, USA.; 2 Department of Surgery, University of Pittsburgh, Pittsburgh, PA 15213, USA.; 3 Department of Bioengineering, University of Pittsburgh, Pittsburgh, PA 15261, USA.; 4 Department of Biochemistry and Molecular Genetics, School of Medicine, University of Colorado Anschutz Medical Campus, Aurora, CO 80045, USA.; 5 Department of Cardiothoracic Surgery, UPMC, Pittsburgh, PA 15213, USA.; 6 Department of Medicine, Division of Gastroenterology, Hepatology and Nutrition, UPMC, Pittsburgh, PA 15213, USA.; 7 Department of Chemical Engineering, Department of Mechanical Engineering and Materials Science, University of Pittsburgh, Pittsburgh, PA 15261, USA.

## Abstract

Chronic inflammatory gastric reflux alters the esophageal microenvironment and induces metaplastic transformation of the epithelium, a precancerous condition termed Barrett’s esophagus (BE). The microenvironmental niche, which includes the extracellular matrix (ECM), substantially influences cell phenotype. ECM harvested from normal porcine esophageal mucosa (eECM) was formulated as a mucoadhesive hydrogel, and shown to largely retain basement membrane and matrix-cell adhesion proteins. Dogs with BE were treated orally with eECM hydrogel and omeprazole (*n* = 6) or omeprazole alone (*n* = 2) for 30 days. eECM treatment resolved esophagitis, reverted metaplasia to a normal, squamous epithelium in four of six animals, and downregulated the pro-inflammatory tumor necrosis factor–α^+^ cell infiltrate compared to control animals. The metaplastic tissue in control animals (*n* = 2) did not regress. The results suggest that in vivo alteration of the microenvironment with a site-appropriate, mucoadhesive ECM hydrogel can mitigate the inflammatory and metaplastic response in a dog model of BE.

## Teaser

Oral delivery of esophageal extracellular matrix hydrogel mitigates mucosal inflammation and metaplasia in a dog model of reflux.

## INTRODUCTION

The phenotype and three-dimensional (3D) organization of cells are strongly influenced by the composition, ultrastructure, and mechanical properties of the extracellular matrix (ECM) (*1*, *2*). Similarly, the composition, ultrastructure, and mechanical properties of the ECM are largely determined by the cell secretome (*1*). This bidirectional, ever-present cross-talk between cells and the surrounding ECM has been aptly named “dynamic reciprocity” (*1*, *2*). In vitro studies show that ECM harvested from site-specific (homologous) tissues can preferentially maintain tissue-specific cell phenotype (*3*), promote cell proliferation (*4*), induce tissue-specific differentiation (*5*), and enhance the chemotaxis of lineage-directed progenitor cells (*6*). The tissue organization field theory (TOFT) extends this concept to factors that influence normal tissue and organ development and homeostasis, the response to injury and environmental stressors, and the initiation and progression of cancer (*7*).

Disease states such as chronic inflammation and neoplasia are associated with changes in intracellular signaling pathways that, in turn, influence cell morphology, function, and the secreted matrisome (*8*). It can be debated whether changes in the ECM are the cause or effect of normal versus abnormal cell phenotype, but regardless, a body of literature now exists that attempts to define a tumor microenvironment (*9*) by its associated cellular RNA profile, gene expression profile, and, more recently, its matrisome (*10*). Specific components of the tumor microenvironment such as COL11A1, fibroblast activation protein (α-FAP), and COX-2 have been suggested as both diagnostic/prognostic markers for the presence or progression of neoplasia (*11*) and as potential therapeutic targets (*12*). Among the tissues and organs investigated with respect to the relationship between ECM microenvironment and development and progression of neoplasia are the ovary (*13*), breast (*14*), and esophagus (*15*–*17*).

The pathogenesis of esophageal adenocarcinoma (EAC) has been well studied and involves progressive dysplastic and metaplastic changes within the esophageal mucosa in response to the recurring insult inflicted by gastric reflux (i.e., stomach acid and bile salts) and the associated chronic inflammation (*18*). Intestinal metaplasia of the esophageal mucosal epithelium, referred to as Barrett’s esophagus (BE), affects approximately 1 to 6% of the U.S. population (*18*, *19*) and is associated with an 11- to 25-fold increased risk of developing EAC (*20*, *21*). A recent study showed that in vitro exposure of metaplastic and neoplastic esophageal epithelial cells to a soluble form of normal esophageal ECM induced a marked down-regulation of cancer-associated molecular pathways and normalization of cell phenotype (*22*). Although cell-matrix cross-talk has been repeatedly shown to affect cell phenotype in vitro, the application of this principle as an in vivo therapeutic approach has not been investigated extensively, primarily because of the inability to affect a change in the local ECM. The present study investigated the in vivo effect of an ECM hydrogel derived from normal porcine esophageal mucosa and composed of the natural constituents of this tissue ECM upon the phenotype of metaplastic esophageal epithelium (BE) in a dog model.

## RESULTS

### Overview of experimental design

**Fig. 1:**
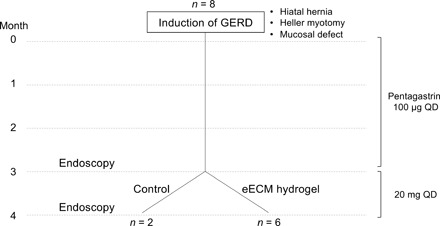
Study overview. A total of eight dogs underwent a reflux-inducing procedure in which a hiatal hernia, a Heller myotomy, and a mucosal defect were created. Animals received pentagastrin daily to increase the acidity of gastric secretions for 3 months. Following pentagastrin administration, animals were evaluated by endoscopy, and pentagastrin was replaced with omeprazole, a PPI commonly used to decrease the acidity of gastric secretions. Animals were randomly divided into two groups: (i) control (*n* = 2), to evaluate the effect of removing pentagastrin and starting omeprazole, and (ii) eECM treatment plus omeprazole (*n* = 6) for 30 days. The macroscopic, microscopic, and clinical outcomes of each eECM treatment animal were compared to pretreatment values for the same animal and to outcomes in the two animals in the control group. QD, one time a day.

A study was conducted to evaluate the effect of a hydrogel composed of ECM derived from normal porcine esophageal mucosa upon the metaplastic epithelium in a dog model of BE. BE was created in eight dogs by modification of a previously described surgical method that results in chronic gastric reflux (*23*, *24*). Treated dogs (*n* = 6) were administered eECM hydrogel orally, twice daily for 30 days. The remaining two dogs were left untreated, with the exception of oral omeprazole, a proton pump inhibitor (PPI), which all eight dogs received. The dogs were euthanized after 30 days of treatment and evaluated by endoscopic, macroscopic, microscopic, and proteomic methods (Fig. 1).

### Viscoelastic properties of ECM hydrogel can be tailored by ECM concentration

**Fig. 2:**
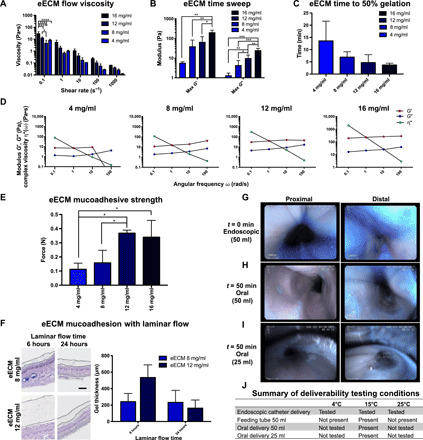
eECM hydrogel viscoelastic and mucoadhesive properties are concentration dependent. Rheological analysis was performed (*n* = 3, means ± SD). (**A**) eECM pre-gel viscosity at 10°C. (**B**) eECM hydrogel maximum storage modulus (*G*′), loss modulus (*G*″), and (**C**) time to 50% gelation after temperature was raised to 37°C. (**D**) Representative graphs of the storage modulus, loss modulus, and complex viscosity η*(ω) of the eECM hydrogels at 37°C, plotted over angular frequency. The ex vivo mucoadhesion of eECM hydrogel to esophageal mucosa was measured by (**E**) tensile testing (*n* = 3, means ± SD) and (**F**) after laminar flow of water for 6 and 24 hours. Gel thickness (H&E staining, black dotted lines) after laminar flow was quantified (*n* = 3, means ± SEM). Scale bar, 500 μm. eECM hydrogel (12 mg/ml) was dyed blue, brought to a temperature of 15°C, and delivered in vivo to the canine proximal and distal esophagus: (**G**) using an endoscopic catheter (50 ml, *t* = 0 min), (**H**) orally (50 ml, *t* = 50 min), and (**I**) orally at the volume used in the animal study (25 ml, *t* = 50 min). Photo credit: Juan Diego Naranjo, University of Pittsburgh. (**J**) Summary of the in vivo deliverability testing conditions at the different ECM hydrogel temperatures. Viscosity: *P ≤ 0.05, ****P ≤ 0.0001 by two-way ANOVA and post hoc Tukey’s test. Storage and loss modulus: *P ≤ 0.05, **P ≤ 0.01, ***P ≤ 0.001 by one-way ANOVA and post hoc Tukey’s test. Mucoadhesive strength: *P ≤ 0.05 by one-way ANOVA and post hoc Tukey’s test.

Rheologic characterization of solubilized ECM (i.e., pre-gel) derived from healthy porcine esophageal mucosa (eECM) was conducted at four concentrations (4, 8, 12, and 16 mg/ml). The concentration was defined as milligrams of dry weight eECM per milliliter of pepsin buffer. The results of rheologic testing were used to determine a preferred formulation that could be delivered orally and endoscopically to the esophagus (Fig. 2, A to D).

eECM pre-gels at each concentration tested were “shear thinning,” defined as a decreased viscosity with increasing shear rate (Fig. 2A). Shear thinning is a property that facilitates the injection of the pre-gel through an endoscopic catheter. Viscosity increased as a function of eECM concentration at the lowest shear rate tested (0.1 s^−1^) by two-way analysis of variance (ANOVA) and post hoc Tukey’s multiple comparisons test (*n* = 3). Viscosity of the eECM pre-gels at the shear rates tested (0.1 to 1000 s^−1^) were all suitable for injection.

Samples were allowed to gel by rapidly raising the temperature to 37°C, and the storage and loss modulus were measured by applying a small oscillatory strain of 0.5% at a frequency of 1 rad/s. eECM hydrogels showed an increased maximum storage modulus (“stiffness”) as the ECM concentration increased. For example, eECM hydrogels at 16 mg/ml had a higher maximum storage modulus (*G*′) compared to 4 mg/ml (*P* = 0.003), 8 mg/ml (*P* = 0.010), and 12 mg/ml (*P* = 0.03) by one-way ANOVA and post hoc Tukey’s multiple comparisons test (Fig. 2B). Similarly, eECM hydrogels at 16 mg/ml had a higher maximum loss modulus (*G*″) compared to 4 mg/ml (*P* = 0.0003), 8 mg/ml (*P* = 0.0004), and 12 mg/ml (*P* = 0.007), and eECM hydrogels at 12 mg/ml had a higher *G*″ compared to 4 mg/ml (*P* = 0.008) and 8 mg/ml (*P* = 0.04). eECM at 12 mg/ml had a stiffness (*G*′) of 67 ± 56 Pa (mean ± SD, *n* = 3). The reported stiffness of normal esophageal mucosa-submucosa is 90 to 240 Pa (*25*).

Gelation time was dependent on ECM concentration (Fig. 2C). At 12 mg/ml, the average time to 50% gelation (i.e., time to reach a storage modulus that was half of its maximum value) was 4.7 ± 3.2 min (means ± SD, *n* = 3). The time of catheter-based delivery can be manipulated wherein the pre-gel does not form a gel within the endoscopic catheter at 37°C, but gels sufficiently quickly to cover the esophageal mucosa before passage into the stomach.

eECM hydrogels at concentrations greater than 4 mg/ml were “stably formed,” defined as the moduli being weakly dependent on frequency, and hence the complex viscosity varies inversely with frequency (*26*). The *G*′ storage modulus was approximately 10-fold greater than the *G*″ loss modulus over the angular frequencies (ω) tested (0.1 to 100 rad/s) for 8, 12, and 16 mg/ml, and for 4 mg/ml at low angular frequencies (≤10 rad/s) (Fig. 2D).

### eECM hydrogel has concentration-dependent mucoadhesive strength ex vivo

Ex vivo mucoadhesive testing was performed to determine the adherence of eECM hydrogel to the esophageal mucosa. Mucoadhesive strength showed a main effect of eECM concentration (*F*_3,8_ = 8.06, *P* = 0.008) by one-way ANOVA (*n* = 3) (Fig. 2E). There was an increase in mucoadhesive strength for eECM as the concentration increased from 4 to 12 mg/ml (*P* = 0.02), 4 to 16 mg/ml (*P* = 0.03), and 8 to 12 mg/ml (*P* = 0.04) by the post hoc Tukey’s multiple comparisons test. There was no difference in mucoadhesive strength for eECM hydrogel between 12 and 16 mg/ml (*P* = 0.97).

### eECM hydrogel adheres to esophageal mucosa with laminar flow for at least 24 hours

To determine whether eECM hydrogel would adhere to the esophageal mucosa in the presence of continuous laminar fluid flow that simulated physiological conditions (e.g., swallowing), eECM hydrogel at two intermediate concentrations (8 and 12 mg/ml) was placed on the mucosal surface of porcine esophagus ex vivo and water was flowed continuously across the surface at 70 ml/min. eECM at 8 and 12 mg/ml was still present on the mucosa at both the 6- and 24-hour time points following initiation of water flow. The amount of gel remaining did not differ between either the two ECM concentrations or the two time points by two-way ANOVA and post hoc Tukey’s multiple comparisons test (*n* = 3 and 6 technical replicates) (Fig. 2F).

The rheology and ex vivo mucoadhesion test results suggested that in vivo delivery and adherence of eECM hydrogel to the esophageal mucosa was feasible. eECM at 12 mg/ml was selected as the preferred hydrogel formulation for in vivo testing due to the increased mucoadhesive strength compared to the other tested concentrations and the relatively fast gelation time (~5 min).

### eECM hydrogel adheres to the esophageal mucosa after in vivo oral and endoscopic delivery

Deliverability testing was adapted from a previously reported method (*27*) and was evaluated in healthy dogs. A blue-colored dye consisting of water, propylene glycol, E133, and 0.1% propylparaben was added to the eECM hydrogel that was then delivered to dogs by (i) an endoscopic catheter or (ii) oral administration. The effects of temperature and volume of the delivered eECM hydrogel were also evaluated.

The eECM hydrogel proved to be deliverable through an endoscopic 5 French (Fr) catheter at 4°, 15°, and 25°C. Representative images for 15°C are shown in the proximal and distal esophagus at the time of application (*t* = 0 min) (Fig. 2G). Oral delivery of the eECM hydrogel was administered with the use of a feeding tube that extended just past the oropharynx or with a catheter tip syringe. Oral delivery of the hydrogel with a feeding tube showed that the hydrogel was present at 15°C but not at 4° or 25°C, and this temperature was used for future experiments. When an initial delivery volume of 50 ml (Fig. 2H) or 25 ml (Fig. 2I) of eECM hydrogel was administered orally at 15°C with a catheter tip syringe, the hydrogel was clearly present at the proximal and distal esophagus for at least 50 min after delivery. Higher retention of the hydrogel was observed at the distal part of the esophagus (Fig. 2, H and I). A summary of the deliverability testing conditions and results is shown (Fig. 2J). Oral delivery of 25 ml of eECM hydrogel (12 mg/ml), twice daily, was selected for the animal study based on the rheology, mucoadhesion, and deliverability experiments.

### Proteomic signature of eECM shows retention of structural, basement membrane, and matrix-cell attachment proteins

**Fig. 3:**
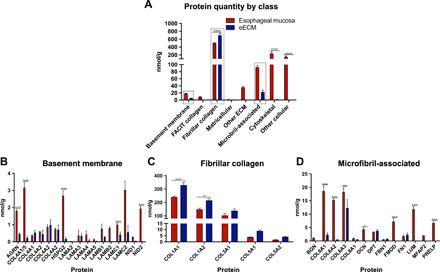
Proteomic signature of eECM. (**A**) Absolute quantification of ECM proteins, binned by gene ontology class, for native esophageal mucosa tissue and eECM. Data are means ± SEM (*n* = 3). Top classes of proteins that showed retention of ECM proteins in eECM compared to native esophageal mucosa tissue included (**B**) basement membrane, (**C**) fibrillar collagens, and (**D**) microfibril-associated proteins (boxed). Proteins within the gene ontology classes are further shown (B to D). *P ≤ 0.05, **P ≤ 0.01, ***P ≤ 0.001, ****P ≤ 0.0001 by two-way ANOVA for treatment and protein functional class or protein type, with post hoc multiple comparisons test.

Quantitative targeted proteomics was performed on eECM and the native esophageal tissue. The eECM was predominantly composed of the fibrillar collagens, including types I, III, and V, which contribute to the physical characteristics of the source tissue (Fig. 3, A and C, and table S1). Fibrillar collagens were enriched compared to native tissue by dry weight (*P* < 0.0001) in the present study (Fig. 3, A and C), as has been shown for other types of ECM (*28*). Notably, there was no difference between eECM and native esophageal tissue for the functional classes of basement membrane proteins (*P* = 0.99) or microfibril-associated proteins (*P* = 0.07) (Fig. 3A). Specifically, there was retention of basement membrane proteins that participate in cell-matrix adhesion such as laminin, perlecan, nidogen, agrin, and collagen type IV (Fig. 3, A and B, and table S1) and microfibril-associated proteins that play an important role in anchoring cells to the collagen-rich matrix such as fibronectin 1, dermatopontin, and fibrillin-1 (Fig. 3, A and D, and table S1). Although select proteins were differentially retained in the matrix (Fig. 3, B to D), the absolute quantity required in vivo is not known. Matricellular proteins as well as structural proteins that contribute to microfibrillar and elastic fiber formation were still present but almost an order of magnitude lower in concentration in the eECM when compared to the source esophageal tissue (table S1). Decellularization of the source tissue resulted in an efficient removal of cellular material (*P* < 0.001) (Fig. 3A), in agreement with a previous report (*29*); specifically, histone 1 (H1), histone 2A (H2A), myosin (MYH), and glyceraldehyde-3-phosphate dehydrogenase were no longer detected, and actin B (ACTB), lamin-A/C (LMNA), vimentin (VIM), and tubulin (TUBB) were present but at an average concentration decrease of 98.91% (table S1).

### Clinical signs of reflux and esophageal intestinal metaplasia occur in the canine model

After reflux inducing surgery, animals showed progressively severe macroscopic esophagitis up to the time of biopsy collection, 90 days after the initial surgery (fig. S1). Three of eight animals showed episodes of anorexia and/or emesis during the 90-day postoperative period. Pentagastrin administration was suspended temporarily for these three animals and then reinstituted after the animals recovered. Two animals required surgical intervention to repair hernia-related complications, i.e., type IV diaphragmatic hernia. One animal developed a bleeding gastric ulcer that was treated with sucralfate, ranitidine, and temporary suspension of pentagastrin. All dogs recovered without further complications.

Gastric acid reflux was monitored before surgery and 2 weeks after surgery by three methods: esophageal intraluminal pH readings, pH reflux events, and impedance reflux events. The pH readings were averaged over 1 hour. All animals showed a decrease in pH after surgery compared to baseline, except for two dogs (C-1 and E-4) (fig. S2A). Reflux was measured with the pH probe for 1 hour and only one animal showed a reflux event preoperatively (C-1), and four animals showed one event postoperatively (E-1, E-2, E-5, and E-6) (fig. S2B). None of the animals showed any impedance events at baseline. Four of the eight animals had one impedance event after surgery (E-1, E-2, E-5, and E-6) (fig. S2C). All animals showed at least one positive reflux reading 2 weeks after surgery during the 1-hour monitoring period, except for C-1.

Animals were subjected to an endoscopic examination to evaluate the gastroesophageal junction (GEJ) and lower esophagus approximately 90 days after surgery. Macroscopic evaluation was performed, and biopsies were collected at this time. Endoscopy was delayed beyond the 90-day time point to as long as 124 days after surgery if the animal had postsurgical complications that resulted in temporary suspension of pentagastrin. Biopsies were taken from areas that showed the greatest severity of disease in all animals. The proximal border of the stomach (z line) was identified before biopsy collection, and macroscopic evaluation around the area of placement of the forceps was performed by the surgeon to ensure that no gastric tissue was collected. To evaluate the consequences of chronic reflux, biopsies were taken from the distal esophagus (fig. S2D). Representative images show the changes in cell phenotype in this canine surgical model: Metaplastic epithelium (fig. S2E) and squamous atypia (fig. S2F) could be seen in biopsies taken from areas that showed macroscopic esophagitis (fig. S2D), and areas that appeared normal by endoscopic visualization showed normal squamous epithelium upon histologic evaluation (fig. S2G).

**Fig. 4:**
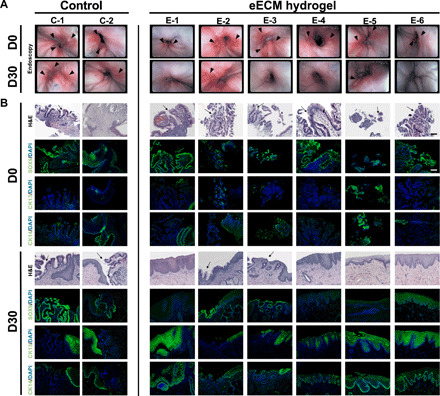
Effect of eECM hydrogel treatment on the macroscopic appearance of the mucosa and esophageal epithelial cell phenotype after 30 days. (**A**) Endoscopies of the lower esophagus/GEJ taken at D0 (before treatment) and at D30 (after treatment) for control and eECM hydrogel animals. Dogs had varied degrees of esophagitis and ulceration (arrowheads) before treatment. No improvement was seen in the animals that only received omeprazole (control). Improvement was seen in all animals after 30 days of eECM hydrogel treatment, including some with complete macroscopic resolution (E-1, E-3, E-4, and E-6). For the remaining treatment animals (E-2 and E-5), there was partial or total resolution of esophagitis with partial healing of the biopsy site. Photo credit: Juan Diego Naranjo, University of Pittsburgh. (**B**) Biopsies were taken before treatment (D0), and location-matched samples were collected after 30 days of treatment (D30) for control animals and eECM hydrogel–treated animals. Samples at D0 and D30 were stained with hematoxylin and eosin (H&E), Barrett’s marker Sox9, or normal esophageal squamous epithelial markers CK13 and CK14. Arrows indicate goblet cells characteristic of intestinal metaplasia and BE. Scale bar, 100 μm.

Endoscopic evaluation showed that all dogs developed esophagitis, which differed in severity between animals before initiating hydrogel treatment at day 0 (D0) (Fig. 4A). Of the control animals, C-1 showed areas of reddened mucosa with occasional erosion, while C-2 had a small area of erosion. E-1 and E-6 had nonconfluent reddish areas in the esophageal mucosa with no apparent signs of ulceration. E-2, E-3, E-4, and E-5 all had areas of ulceration in the esophageal mucosa. E-3 and E-4 also had scar tissue present. E-2 showed the most severe changes of all the dogs with ulcers and erythema that involved approximately 60% of the esophageal circumference.

Histologic analysis of biopsies at D0 showed the presence of columnar metaplastic cells in seven of eight dogs; all but C-2 (Fig. 4B). C-2 showed atypia but no metaplasia of the normal stratified squamous epithelium.

### eECM hydrogel mitigates macroscopic esophageal inflammation

The two dogs randomized to receive omeprazole only (i.e., no eECM hydrogel) (C-1 and C-2) showed continued presence of esophagitis upon endoscopic examination (redness or areas of erosion) after 30 days of treatment (D30) (Fig. 4A). In contrast, four of six eECM hydrogel-treated dogs (E-1, E-3, E-4, and E-6) showed near-complete resolution of macroscopic esophagitis after 30 days of treatment compared to D0. The remaining two eECM hydrogel–treated dogs (E-2 and E-5) showed improvement but did not show resolution of macroscopic esophagitis after 30 days of treatment (Fig. 4A).

### eECM hydrogel mitigates metaplastic change in the esophageal mucosa

Histologic evaluation of the hematoxylin and eosin (H&E)–stained slides and the slides immunolabeled for normal differentiated epithelial cell markers (suprabasal CK13^+^/basal CK14^+^), Barrett’s markers (Sox9^+^) (Fig. 4B), and (Alcian blue + goblet cells) (fig. S3) was conducted on pretreatment biopsies (D0) and posttreatment location-matched samples (D30). A lower magnification of the same areas is shown in fig. S4, and a histologic overview of the esophageal circumference at D30 is shown in fig. S5.

At D0, seven of eight dogs, all but C-2, showed intestinal metaplasia of the esophageal epithelium (Fig. 4B). The metaplastic tissue was Sox9^+^, with minimal expression of CK13^+^/CK14^+^. C-2 had Sox9^−^ atypical epithelium at D0.

The majority of the esophageal mucosa in dog C-1 was still metaplastic (Sox9^+^) with scattered foci of normal epithelium (CK13^+^/CK14^+^) after 30 days of omeprazole treatment (Fig. 4B). C-2 did not show metaplasia at D0 but then developed an area of Sox9^+^ metaplasia, with adjacent areas of normal epithelium (CK13^+^/CK14^+^) after 30 days of omeprazole treatment (Fig. 4B).

In contrast, four of the six eECM-treated dogs (E-1, E-4, E-5, and E-6) showed histologic reversion of the metaplastic esophageal epithelium to a normal, differentiated squamous epithelium (CK13^+^/CK14^+^/Sox9^−^) in the same areas where columnar metaplasia (Sox9^+^) had previously been identified (Fig. 4B). E-2 and E-3 did not fully revert to normal epithelium. E-2 showed foci of columnar metaplasia and CK13^+^/CK14^+^ epithelium (Fig. 4B and fig. S5), with areas of stratified squamous epithelium beneath metaplastic goblet cells. E-3 showed largely normal CK13^+^/CK14^+^ epithelium with small focal areas of Sox9^+^ cells (Fig. 4B and fig. S5). Alcian blue stains goblet cells and is commonly used as a secondary marker of BE. Results of the Alcian blue staining corroborated the Sox9^+^ staining results at D0 and D30 for all dogs (fig. S3).

### eECM hydrogel down-regulates TNFα^+^-expressing cells and promotes a cytokine profile similar to normal esophageal tissue

**Fig. 5:**
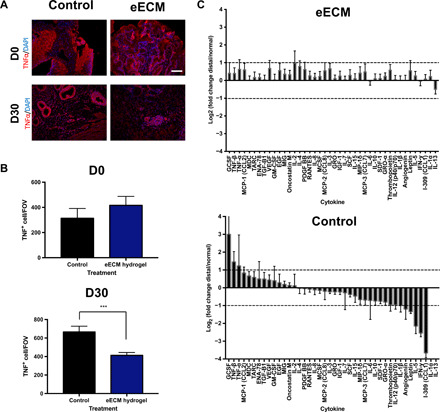
Effect of eECM hydrogel treatment on TNFα^+^proinflammatory cell infiltrate and cytokine expression after 30 days. (**A**) TNFα is a proinflammatory cytokine up-regulated in gastroesophageal reflux disease progression. TNFα immunolabeling with DAPI counterstain was performed on samples collected before and after 30 days of treatment for control and eECM hydrogel–treated animals. Representative images are shown. (**B**) Three pictures per animal were imaged and quantified using CellProfiler and compared to control. Scale bar, 100 μm. Data are means ± SEM for control (*n* = 2) and eECM hydrogel treatment (*n* = 6), with three technical replicates per sample. ***P ≤ 0.001 by Student’s unpaired *t* test. (**C**) Differential expression of cytokines in the eECM hydrogel and control animals’ esophageal tissue after 30 days of treatment, and normalized by the animals’ own proximal, normal esophageal tissue using a cytokine antibody microarray. Differentially expressed cytokines were defined as a >2- or <−2-fold change of eECM treatment or control compared to normal (dashed line).

Immunolabeling to identify tumor necrosis factor–α (TNFα) was performed to determine the presence of a proinflammatory cell infiltrate in both the control and treatment animals at D0 and D30 (Fig. 5A). Three images per sample were examined, and the number of TNFα^+^ cells was quantified.

There was no difference in TNFα^+^ cells for eECM-treated dogs (*n* = 6) compared to control dogs (*n* = 2) at D0 before treatment. TNFα^+^ cells decreased with eECM treatment compared to control after 30 days of treatment (*P* = 0.0007, Student’s unpaired *t* test) (Fig. 5B).

The differential expression of cytokines in eECM-treated animals and control animals was further characterized with a cytokine array. The cytokine values were normalized to each animal’s proximal, normal esophageal tissue (Fig. 5C). Tissue from dogs treated with eECM hydrogel did not show any differences in cytokine values compared to normal esophageal tissue except for interleukin-2 (IL-2; 2.0-fold increase). Tissue from control dogs notably showed an increase in proinflammatory cytokines such as TNFβ (2.7-fold increase) and TNFα (2.3-fold increase) compared to normal esophageal tissue.

Differential expression in cytokine expression became more apparent when the normalized values for eECM-treated animals were compared to the normalized values in control animals, which showed a markedly different pattern of cytokine expression (Fig. 5C). Chemokines, such as MIP-1δ (CCL15) (2.2-fold), MCP-3 (CCL7) (2.3-fold), GRO-α (CXCL1) (2.1-fold), and I-309 (CCL1) (13.6-fold), and tissue remodeling cytokines, such as angiogenin (2.3-fold), leptin (3.8-fold), and thrombopoietin (3.0-fold), were down-regulated in the control animals, suggesting a higher expression in the eECM-treated animals. Normalized values for immune activating granulocyte-colony stimulating factor (6.0-fold) and proinflammatory TNFβ (2.0-fold) were up-regulated in control animals, but not in eECM-treated dogs. However, contrary to our hypothesis, normalized values for proinflammatory cytokines IL-1β (2.0-fold), IL-5 (5.2-fold), IL-12 (2.0-fold), and interferon-γ (5.6-fold) were down-regulated in the control dogs, suggesting a higher expression in the eECM-treated dogs. When the data are considered collectively, animals treated with eECM hydrogel showed a cytokine expression more similar to normal tissue, with higher expression of tissue remodeling cytokines and chemokines compared to control animals, and lower expression of proinflammatory mediators TNFβ and TNFα, results that corroborate the TNFα immunolabeling.

### eECM hydrogel promotes a protein signature similar to normal esophageal tissue, with an increase in basement membrane proteins and matrix-cell adhesion proteins

**Fig. 6:**
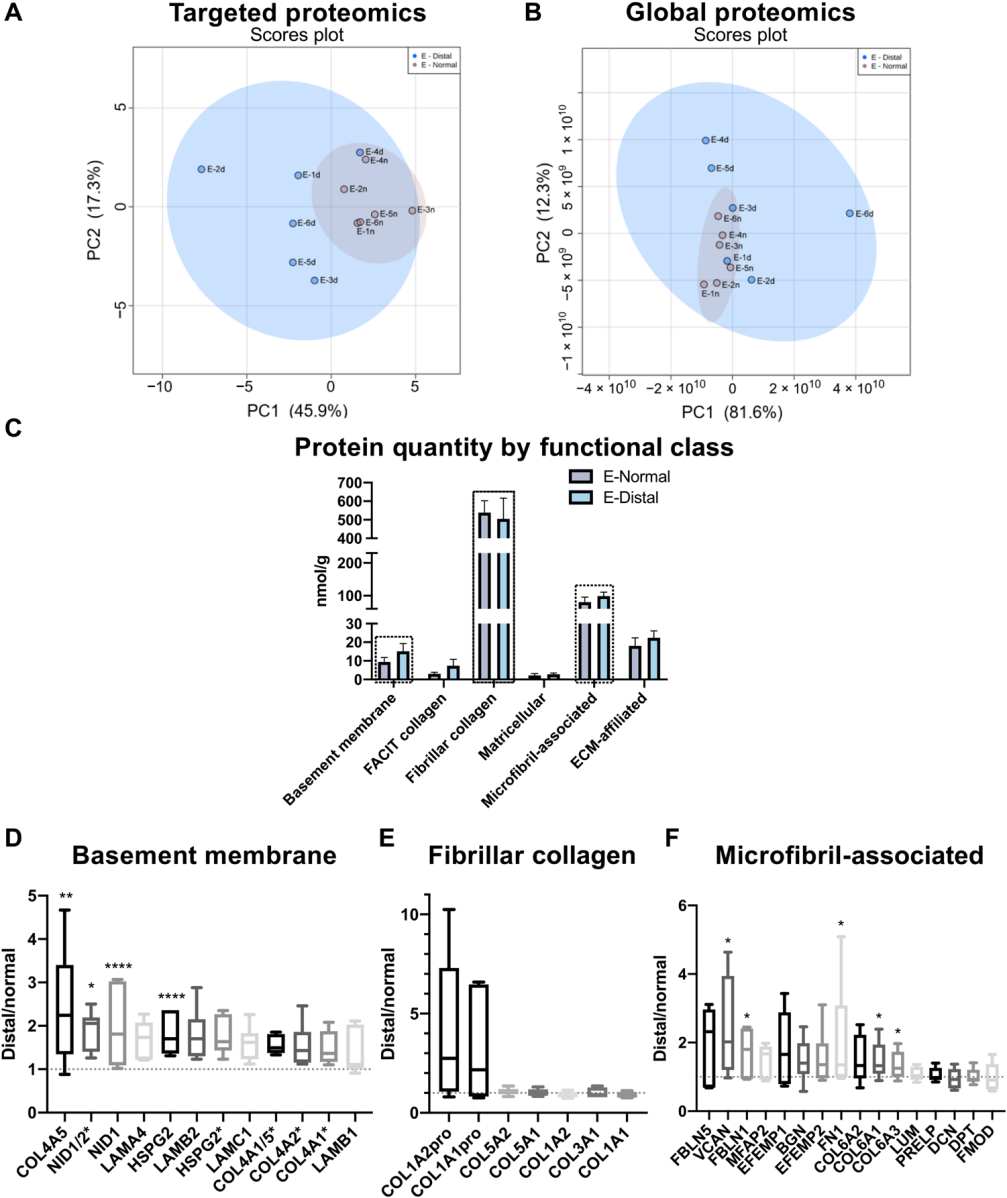
Proteomic signature of eECM-treated tissue. Principal components analysis (PCA) of “distal” tissue and “normal” tissue of eECM-treated dogs at D30 for (**A**) targeted proteomics and (**B**) global proteomics, showing the overlap between the remodeled distal tissue (E-Distal) and normal tissue (E-Normal) (*n* = 6). (**C**) Absolute quantification of ECM proteins, binned by gene ontology class, for distal and normal tissue of eECM-treated dogs at D30. Data are means ± SEM (*n* = 6). Expression of (**D**) basement membrane proteins (**E**), fibrillar collagen proteins, and (**F**) microfibril-associated proteins is expressed as a ratio of distal/normal for individual proteins within each class for the eECM-treated animals at D30. *P ≤ 0.05, **P ≤ 0.01, ****P ≤ 0.0001 by two-way ANOVA for tissue type and protein functional class or protein type, with post hoc multiple comparisons test.

The ECM of the distal esophagus in eECM hydrogel–treated animals was compared to normal esophageal tissue in each dog that had developed columnar metaplasia at D0. One control animal and all six ECM hydrogel–treated animals met this criterion at D30 and were evaluated. The distal, remodeled tissue of eECM hydrogel–treated animals (E-Distal) showed a protein profile that was similar to normal esophageal tissue (E-Normal) in composition based on both targeted (quantitative) ECM proteomics (Fig. 6, A and C, and table S2) and global protein profiles (Fig. 6B). As expected, the tissue samples from dogs treated with eECM hydrogel (E-Distal) showed higher variability than tissue from the normal controls (E-Normal) (Fig. 6, A and B). Overall, however, the protein profile of eECM-treated tissues was not statistically different when comparing functional protein classes to normal esophageal tissue by absolute (quantitative) protein expression (Fig. 6C).

Notably, there was an average 1.6-fold change increase for 14 basement membrane proteins in eECM-treated remodeled tissue compared to normal tissue (Fig. 6, C and D, and table S2). Select basement membrane proteins within the class were increased including collagen α5 (IV) (2.5-fold increase, *P* = 0.005), nidogen 1/2 (1.8-fold increase, *P* = 0.03), nidogen 1 (1.7-fold increase, *P* < 0.0001), and perlecan (HSPG2) (1.8-fold increase, *P* < 0.0001). Several laminin subunits showed a greater than 1.5-fold change (e.g., laminin α4, laminin β2, and laminin γ1) but were not significant. Laminin α3 was found to be retained in the porcine eECM compared to the native tissue (Fig. 3B). The probe was not homologous for canine laminin α3, and therefore this protein was not evaluated.

The class of fibrillar collagens (Fig. 6, C and E, and table S2) showed approximately a ratio (“distal/normal”) of ~1 for fibrillar collagens (I, III, and V) except for an increase in the pro-form of collagen type-1 α1 (2.9-fold increase, *P* = 0.08) and α2 (3.6-fold increase, *P* = 0.06) (Fig. 6E).

Microfibril-associated proteins showed a ratio (distal/normal) of ~1 (Fig. 6, C and F, and table S2) (e.g., lumican, dermatopontin, and decorin) but was increased for proteins such as versican (2.2-fold increase, *P* = 0.03), fibronectin 1 (1.7-fold increase, *P* = 0.04), and fibulin 1 (1.6-fold increase, *P* = 0.045).

In contrast, the comparison of normalized distal tissue in the control animal to normalized esophageal ECM-treated animals showed a different expression pattern [e.g., lower basement membrane protein expression of collagen α1/5 (IV), collagen α5 (IV), laminin β2, and nidogen; and higher collagen α1 (XII), tenascin C, fibulin 5, periostin, and biglycan] (*n* = 1) (table S2).

### Oral administration of eECM hydrogel does not induce adverse systemic or gastrointestinal effects

No episodes of emesis or diarrhea occurred during the treatment period. Animal weight did not change for any of the eECM-treated animals throughout the course of the study (fig. S6, A and B). There were no abnormalities in electrolyte or serum chemistry values before or after treatment (*n* = 3) (fig. S6C).

## DISCUSSION

Results of the present study show that intestinal metaplasia can be induced in the esophageal mucosa as a result of changes in the microenvironment, specifically the induction of chronic gastric reflux, decreased pH, and the associated chronic inflammation in this canine model. Furthermore, the results suggest that the inflammation can be mitigated and the metaplastic changes can be reversed when the microenvironment is returned, at least in part, to a more normal, homeostatic state by coating the esophageal mucosa with a mucoadhesive hydrogel composed of normal porcine esophageal ECM.

The matrisome of the eECM biomaterial was characterized using stable isotope-labeled peptides targeted toward the ECM (“targeted proteomics”) for absolute protein quantification. The eECM retained structural proteins (e.g., collagen types I, III, and V), basement membrane proteins related to matrix-cell signaling (e.g., laminins, perlecan, nidogen, agrin, and collagen type IV), and matrix-cell attachment proteins (e.g., fibronectin 1, dermatopontin, and fibrillin-1) that are present in healthy native porcine esophageal tissue. It is plausible that laminin-311, a basement membrane protein, was retained in the eECM, because of the retention of its heterotrimer subunits α3, β1, and γ1 (Fig. 3B). Laminin-311 forms a complex with perlecan and nidogen-1 (*30*), which were also retained in the eECM in the present study (Fig. 3B), and this complex is considered essential for epithelial cell attachment and basement membrane assembly (*31*). Such complexes are necessary for normal tissue structure and function, and their formation is made possible by the presence of all required individual subunits, a scenario that exists in a hydrogel composed of the full complement of matrix molecules. Such complexes would not be present if the hydrogel was composed of isolated components of ECM such as collagen or laminin. The constructive effects of the eECM hydrogel upon the inflammatory response and epithelial cell phenotype in the present study cannot be definitively attributed to any single component or combination of components. There is a differential and distinctive expression of ECM components in the various segments of the gastrointestinal (GI) tract, and the functional and structural roles of these components during morphogenesis and homeostasis are only partially understood (*32*).

The findings of this in vivo pilot study are consistent with the principles of dynamic reciprocity and the TOFT (*1*, *2*, *7*) and have particular relevance for the metaplastic changes that occur in the esophageal mucosa as a result of chronic gastric reflux and the associated altered microenvironment (*33*). Seven of eight dogs (88%) developed intestinal metaplasia of the esophageal mucosa within 90 days of the onset of gastric reflux in the present study, consistent with previous reports of this model that showed a Barrett’s incidence ratio of 75% (*23*, *24*), and further validates this experimental model. The subsequent exposure of the metaplastic epithelium to a hydrogel form of normal esophageal mucosa ECM combined with daily administration of the PPI omeprazole halted and reversed this metaplastic change in four of six dogs. The metaplastic change in two dogs did not fully revert with eECM treatment. One showed partial reversal to a multilayered epithelium that is considered an intermediate step between gastroesophageal reflux disease (GERD) and Barrett’s disease (E-2) (*34*) and the second showed small foci of columnar epithelium (E-3). None of the six eECM-treated animals showed statistically different protein profiles based on functional classification compared to normal esophageal tissue by ANOVA analysis. In contrast, of the two dogs that received omeprazole only and served as a model control, one dog showed persistence of inflammation and metaplasia, and the second dog progressed from an atypical squamous epithelium to metaplasia. The relative contribution of the eECM matrisome versus the omeprazole to the constructive effects observed in the eECM treatment group cannot be determined from the results of this study. There were no dogs that received the eECM hydrogel alone during the post 90-day treatment period. However, neither of the two control dogs that received omeprazole alone showed any mitigation of the chronic inflammation or abnormal mucosal phenotype, and one of these dogs showed progressive pathology. Although the progression of disease was evaluated by multiple methods including histology and proteomic analysis, the limited *n* value for the control animals and the single time point (30 days following initiation of treatment) of evaluation are limitations of the present study.

Esophageal tissue from animals treated with eECM hydrogel, taken from a distal area that previously showed metaplasia, was compared to autologous normal esophageal tissue after 30 days of treatment, and showed a near-normal protein and cytokine profile, shown as a ratio (distal/normal) ~ 1 (Figs. 5C and 6, D to F). A ratio of 1 is a preferred outcome in a tissue remodeling site, suggesting protein regulation consistent with “homeostasis” (*35*). The classes of proteins and chemokines that were returned to near-normal levels in the remodeled esophageal tissue of eECM-treated animals are indicative of a constructive tissue repair response, which is consistent with the observed histologic reversion of the metaplastic epithelium in four of six animals. Targeted proteomics showed an increase in basement membrane proteins (e.g., collagen type IV, nidogen, perlecan, and laminins) that are known to be essential for epithelial cell differentiation (*36*), microfibril-associated proteins (e.g., versican, fibronectin, and fibulin 1) that have been shown to mediate cellular migration and vasculogenesis (*37*), and procollagens, shown to be associated with increased healing of injured tissues (*38*).

The concept that cell phenotype and morphogenetic patterning are directed, or at least modulated, by microenvironmental cues, including selected ECM molecules, is not new (*1*, *39*). Bissell *et al*. (*1*) described a model of the relationship between the ECM, the cytoskeleton, and gene expression. Kratochwil (*39*) showed that normal breast epithelial cells cultured upon the ECM of salivary gland tissue assumed not only a salivary gland phenotype but also the glandular patterning of salivary gland tissue. These in vitro studies among others have made it clear that factors present in the ECM-based microenvironmental niche are continuously providing signals that modulate cell behavior. This concept had been applied as a therapeutic approach by Hurst *et al*. (*40*) who investigated the effect of ECM hydrogel to suppress tumorigenic growth of bladder cancer cells when coinjected into the flanks of mice. Bladder cancer cells coinjected with ECM hydrogel showed a more differentiated and less atypical phenotype compared to bladder cancer cells coinjected with a neoplastic ECM (Matrigel) control, which displayed a malignant phenotype and tumorigenic growth. The results of the present study suggest that TOFT can be similarly applied in vivo to abnormal esophageal cell phenotype. It is plausible that the eECM hydrogel had a direct effect upon the differentiation state of the metaplastic cells or, alternatively, promoted esophageal stem cell differentiation toward a normal phenotype as has been shown in previous in vitro studies (*5*, *22*). eECM hydrogel has been shown to down-regulate key neoplastic signaling pathways (phosphatidylinositol 3-kinase–Akt, cell cycle, and G1-to-S phase transition) in esophageal OE33 neoplastic cells in vitro, and interestingly, the same signaling pathways were increased in nonmalignant Het-1A epithelial cells (*22*). The observed opposite effects of eECM hydrogel on neoplastic and nonmalignant cells would be advantageous for a therapy delivered to the diseased esophagus. eECM hydrogel was also shown to promote basally polarized, normally differentiated (CK13^+^/CK14^+^) stem cell organoids in 3D culture (*5*). Therefore, it is also plausible that the eECM hydrogel modulated renewing stem cells following reflux injury.

The transformation of both normal esophageal stem cells and differentiated epithelial cells to abnormal metaplastic and dysplastic phenotypes of BE is associated with the chronic presence of proinflammatory cells in Barrett’s pathogenesis (*41*). Specifically, TNFα is a potent proinflammatory cytokine that is up-regulated with GERD-Barrett’s-EAC progression (*42*). The chronic presence of TNFα contributes to multiple mechanisms of Barrett’s disease progression including inducing epithelial cells to proliferate and secrete reactive oxygen species that can lead to DNA damage (*43*), reducing E-cadherin levels/cell-cell adhesions (*44*) that can expose resident stem cells to bile salts and acid (*33*, *41*, *42*), and up-regulating matrix metalloproteinases (*42*). Results of the present study suggest that twice daily oral administration of a mucoadhesive eECM hydrogel mitigates the macroscopic and microscopic inflammatory changes and is associated with a decrease in TNFα inflammatory cells, which was corroborated by the cytokine microarray that showed increased expression of TNFα and TNFβ in the control animals but not in the eECM hydrogel animals. In addition, the eECM hydrogel–treated animals showed a cytokine profile more similar to normal esophageal tissue, compared to the two control animals, and showed a relative increase in chemokines (e.g., MIP-1δ, MCP-3, GRO-α, and I-309) and tissue remodeling cytokines (e.g., angiogenin, leptin, and thrombopoietin), which are known to promote tissue healing.

In vitro and in vivo studies have consistently shown that ECM degradation products, including eECM, promote the transition of a proinflammatory macrophage phenotype toward a regulatory, anti-inflammatory phenotype (*45*). The results of the present study were similar to those of a previous study wherein ECM hydrogel delivered via enema for 1 week down-regulated TNFα^+^/CD68^+^ macrophages in a rat model of ulcerative colitis, another chronic inflammatory-driven disease of the GI tract. The down-regulation in TNFα^+/^CD68^+^ macrophages correlated with the restoration of a near-normal colonic epithelium, restoration of mucosal epithelial barrier function, and increased E-cadherin^+^ cell-cell adhesion (*46*). In summary, eECM hydrogel most likely does not target one aspect of the molecular pathogenesis of metaplasia but rather acts through a “whole microenvironment” approach, such as modulating epithelial cell phenotype and reducing the proinflammatory TNFα^+^ cell infiltrate.

The use of solid sheet forms of ECM for the treatment of esophageal disease has been successful in stricture prevention (*47*) and augmentation of anastomosis at the GEJ (*48*) in large-animal models. ECM bioscaffolds have also been used in humans for patch esophagoplasty (*49*) after mucosal resection of T1a EAC (*50*) and for in vivo regeneration of an esophageal full thickness segmental defect (*51*). In contrast to the above surgical applications of ECM-based surgical meshes, the pilot study described here used a hydrogel form of ECM to change the environmental milieu at the site of metaplastic esophageal epithelium.

From a practical perspective, the success of a therapy delivered to the esophagus is dependent on its ability to reach and remain at the desired location for its bioinductive effect. The material properties of ECM hydrogels can be tailored by varying ECM concentration and temperature (*52*). eECM at 12 mg/ml concentration was selected for preferred characteristics such as having a relatively fast gelation time (~5 min), a stiffness near that of esophageal mucosa, the highest mucoadhesion to the esophageal surface compared to other ECM concentrations, and being stably formed. Furthermore, the pre-gel could be effectively and safely delivered by both endoscopic and oral administration and remain for at least 50 min with a higher amount of hydrogel adhering to the distal esophagus in comparison to the more proximal areas. The amount (thickness) of hydrogel on the esophageal mucosa preferred for therapy is not known, but only the most luminal layer of the hydrogel would be in contact with the esophageal epithelium, and the current delivered volume showed efficacy in four of six animals. An ECM hydrogel composed of porcine cardiac ECM has recently been successfully and safely used for intracardiac injection following myocardial infarction in a phase 1 clinical trial (ClinicalTrials.gov: NCT02305602), showing the potential for clinical translation of eECM hydrogel therapy.

The potential clinical implications of the present study are noteworthy. GERD, BE, and EAC are serious problems that have limited therapeutic options. The current standard of care for these clinical problems is associated with unacceptable morbidity and a continued increase in the incidence of EAC in the absence of other risk factors (*53*). Further studies are required to investigate the efficacy of an ECM hydrogel as a treatment option for the esophageal pathology caused by gastric reflux, but the results of this pilot study are promising and, importantly, based on accepted concepts of cell-matrix interactions.

## MATERIALS AND METHODS

### Study design

The objective of the present study was to determine the effect of eECM hydrogel harvested from normal tissue upon metaplastic cell phenotype in a canine model. Secondary objectives were to determine the appropriate eECM hydrogel formulation for delivery to the esophageal site of interest and the safety of the therapy. Chronic gastric reflux was induced in eight dogs by a reflux-inducing procedure in which a hiatal hernia, Heller myotomy, and a mucosal defect were created, and pentagastrin was injected daily for 90 days to increase the acidity of gastric secretions. The resultant alteration of the microenvironment in the distal esophagus resulted in intestinal metaplasia and squamous atypia of the esophageal mucosa after 90 days. The effect of eECM hydrogel administered orally upon the phenotype of the mucosal epithelium was then determined. It was hypothesized that the eECM hydrogel treatment would mitigate, or revert, metaplastic epithelial cell phenotype, down-regulate proinflammatory cell activation, and be safely tolerated. Data collection time points were determined prospectively: at baseline, after reflux-inducing surgery, before the start of treatment, and after 30 days of treatment at which time the animals were euthanized (Fig. 1). Animals served as their own control before and after treatment. All data were shown; no outliers were found or excluded. The technical and biological replicates are defined for each test, with at least *n* = 3. Animals were randomly assigned to treatment groups at the start of the study and before any procedures were performed. It was not possible to blind investigators during the treatment of the animals. The pathologist was blinded to the treatment group and time point at which the specimens were collected.

### ECM hydrogel preparation

eECM was prepared by decellularizing porcine tissue as previously described (*5*, *29*). eECM was solubilized using pepsin (1 mg/ml) in 0.01 M HCl, in a 10:1 ratio of ECM/pepsin, for 48 hours at room temperature (“ECM digest”) (*26*). The ECM digest was stored at −20°C. Samples were thawed at 4°C and neutralized to physiologic pH and salt concentration with 0.1 M NaOH (1:10 volume of pre-gel solution) and 10× phosphate-buffered saline (PBS) (1:9 volume of pre-gel solution). The digest was diluted to the desired ECM concentration (4, 8, 12, and 16 mg/ml) with 1× PBS at 4°C (“eECM pre-gel”) (*26*). The temperature was increased to 37°C for gelation to occur (“eECM hydrogel”).

### Rheology

The viscoelastic properties of the eECM pre-gel and formed eECM hydrogel were determined with a temperature-controlled, 40-mm parallel plate rheometer (AR2000) as previously described (*54*) and according to the American Society for Testing and Materials Standard F2900-11. The pre-gel form was tested at four concentrations (4, 8, 12, and 16 mg/ml) (*n* = 3) to determine a preferred formulation for oral and/or endoscopic delivery to the esophagus. eECM concentration is a factor that can contribute toward hydrogel viscoelastic properties (*52*).

eECM digests were neutralized and kept at 4°C (“pre-gel”) and tested within a 2-hour window. The pre-gel samples were loaded onto the AR2000 rheometer (TA Instruments) with a parallel plate geometry precooled to 10°C, a temperature well below gelation. Mineral oil was used to seal the sample plate interface and to minimize evaporation during the testing. A series of rheological tests were conducted for each sample in sequence. A steady-state flow curve at 10°C was performed to determine the viscosity profile of the eECM pre-gel solution at a range of shear rates (0.1 to 1000 s^−1^). Plate temperature was rapidly raised from 10° to 37°C to induce gelation, and an oscillatory time sweep was performed at 37°C, by applying a small, 0.5% oscillatory strain at a frequency of 1 rad/s to measure the maximum storage modulus (*G*′), maximum loss modulus (*G*″), and gelation kinetics of the fully formed eECM hydrogel (occurring within 40 to 60 min). The time to 50% gelation was measured as the time to 50% of the maximum storage modulus. An oscillatory frequency sweep at 37°C was performed after gelation was complete to determine the storage modulus (*G*′), loss modulus (*G*″), and complex viscosity η*(ω) over a range of angular frequencies (ω) (0.1 to 100 rad/s) by applying a small, 0.5% oscillatory strain. Data were extracted using the Rheology Advantage Data Analysis software (version 5.7, TA Instruments, New Castle, DE) and exported to Prism (version 6, GraphPad) for statistical analysis.

### Mucoadhesion

#### Mucoadhesive strength

A custom-designed test was used to measure mucoadhesion of the eECM hydrogel to porcine mucosa ex vivo. Porcine esophageal mucosa was mechanically isolated from the underlying muscularis layer. Four milliliters of eECM pre-gel at four ECM concentrations (4, 8, 12, and 16 mg/ml) (*n* = 3) were placed at the bottom of each well of a six-well plate. Porcine esophageal mucosa was glued to the outer convex surface of a half-sphere (40 mm diameter). The temperature was increased to 37°C to cause ECM hydrogel formation and adherence to the esophageal mucosa. The construct was then placed on the MTS Insight Tensile machine with 10-N load cell and ball burst attachment, set to a measuring frequency of 10 Hz. The ball burst attachment was securely attached to the half-sphere, and the half-sphere was raised up at a rate of 5 mm/min until separation of the hydrogel from the mucosa occurred. The maximum force value was considered the adhesion strength. Measurements were only accepted if the detachment occurred between the mucosa and the hydrogel.

#### Mucoadhesion with laminar flow

The intermediate ECM concentrations (8 and 12 mg/ml) of eECM were selected for mucoadhesion testing with laminar flow ex vivo. A thin layer of eECM pre-gel (2 ml) was spread evenly across a porcine esophageal mucosal surface cut to specifications 6.5 cm long × 2.5 cm wide. The gel-mucosa surface was placed in a flow chamber and immediately subjected to flow of type I water at 70 ml/min at 37°C. The test was performed for 6 or 24 hours (*n* = 3); the sample was then formalin-fixed, stained with H&E, and imaged with a bright-field microscope; and the thickness of remaining gel was quantified by ImageJ with six technical measurements per sample.

### Targeted mass spectrometry

Native porcine esophageal mucosa tissue and eECM were prepared for proteomic analysis of the biomaterial (*n* = 3). Similarly, biopsy punches of normal and distal esophageal mucosa tissue from each dog were taken at the time of necropsy; half of each sample was flash-frozen for proteomic analysis of the remodeled tissue site, and half was formalin-fixed for histologic confirmation of the tissue disease state. The canine tissue for proteomic analysis met the following criteria: (i) in an area that was metaplastic at D0 (by biopsy assessment) and (ii) definitively in the esophagus (non-GEJ tissue). Six eECM-treated animals and one control animal met these criteria. Esophageal samples were processed as previously described (*28*). Briefly, fresh frozen samples were milled in liquid nitrogen with a mortar and pestle and lyophilized to dryness. Approximately 2.5 mg of samples was processed by stepwise extraction, resulting in a cellular, soluble ECM, and insoluble ECM fraction for each sample. Protein concentration was determined by A660 Protein Assay (Pierce). Enzymatic digestion was carried out by filter-aided sample processing (*55*) with 10-kDa molecular weight cutoff filters. Thirty micrograms of proteins from each sample was added to the filter in combination with 500 fmol of stable isotope-labeled quantitative concatemers (*56*) representing ECM, ECM-associated, and cellular proteins of interest (*57*). Samples were then reduced, alkylated, and digested with trypsin at 37°C for 14 hours. Peptides were recovered with successive washes of ammonium bicarbonate and 0.1% formic acid, dried, and brought up to final volume for liquid chromatography–mass spectrometry (LC-MS) injection.

Quantitative analysis of the ECM was carried out by LC-selected reaction monitoring (LC-SRM) analysis on a QTRAP 5500 triple quadrupole mass spectrometer (AB Sciex) coupled with an ultrahigh performance LC (UPLC) Ultimate 3000 (Thermo Fisher Scientific) with acquisition methods as previously described (*58*). Each sample was injected and separated by reversed-phase chromatography (Waters, Acquity UPLC BEH C18, 1.7 μm 150 × 1 mm) by running a gradient from 2 to 28% acetonitrile in 0.1% formic acid for 28 min at a flow rate of 150 μl/min. The MS was run in positive ion mode with the following settings: source temperature of 210°C, spray voltage set to 5300 V, curtain gas of 20 psi, and source gas of 35 psi (nitrogen gas). Data were acquired using the instrument-controlled software, Analyst (v1.6.2). QconCAT transition selection, declustering potential, collision energies, and retention times were specifically optimized for each peptide of interest using Skyline’s software (*59*).

Data analysis for LC-SRM runs was carried out in the Skyline software package. Briefly, peaks were manually validated and light to heavy ratios (^12C^_6_^/13C^_6_) for each target peptide were used to back calculate the concentration of each endogenous peptide targeted in the sample.

### In vivo deliverability testing

All animal procedures were approved by the University of Pittsburgh Institutional Animal Care and Use Committee. Eight healthy female mongrel dogs 6 to 8 months old (Marshall BioResources, Wayne County, New York) weighing approximately 20 kg were used in the present study. The ability of the hydrogel to be injected through a 12-Fr catheter and to adhere and be retained on the esophageal mucosa in vivo was evaluated in a canine model. The method was previously used in humans to test deliverability of different hydrogels to the esophageal mucosa (*27*). In brief, the neutralized hydrogel was colored with a mixture of water, propylene glycol, E133, and 0.1% propylparaben blue dye for 9 hours with constant stirring at 4°C to prepare for administration. Animals were given 0.25 ml of acepromazine for mild sedation, and 25 or 50 ml of 12 mg/ml neutralized hydrogel was delivered at 4°, 15°, and 25°C either orally, with the use of a 12 Fr × 16″ feeding tube (Medtronic), which was placed in the proximal esophagus, or by placing a 60-ml catheter tip syringe (Monoject, Covidien) at the mouth of the animal. After delivery of the hydrogel, animals were denied access to any food or water until start of the endoscopic procedure. Each dog was anesthetized by induction with ketamine (5 to 11 mg/kg) for the endoscopic procedure, and surgical plane anesthesia was maintained with 1 to 5% isoflurane via endotracheal tube. The animal was placed in sternal recumbency with the neck extended to avoid pressure from the endoscope on the trachea or the nearby vessels. If necessary, insufflation with minimal amounts of air was done to facilitate appropriate visualization. The animal was placed at a 50° angle with the head lifted upward. The endoscope was advanced slowly to identify the hydrogel.

For evaluation of endoscopic delivery of the hydrogel, an EDC190 delivery catheter (MILA International) 5 Fr was used. The gel at the desired temperature was injected onto the esophageal surface. Video recording was performed throughout the procedure.

### Intraesophageal pH monitoring

Intraesophageal pH monitoring was performed on all animals before surgery at baseline and 6 weeks after the reflux inducing surgery. The procedure was performed on fasted animals. The positioning of the pH probe (Ohmega pH Impedance recorder, Laborie) was done while the animals were awake. The position of the probe was determined by advancing the probe into the stomach until an acid readout was obtained. The probe was then withdrawn until the readout became alkaline, and, from this point, it was further withdrawn 5 cm to its final position. The reading was registered during a 1-hour period. Animals were presedated with acepromazine, and low-dose ketamine (5 to 11 mg/kg) was used at the moment of placing the probe.

### Reflux inducing surgical procedure

An overview of the animal model is presented (Fig. 1). A modified version of the model developed by Hennessy *et al*. (*23*, *24*) was used. All animals were subjected to surgery to induce reflux by (i) creating a hiatal hernia and (ii) performing a Heller myotomy to create functional insufficiency of the lower esophageal sphincter.

Each dog was anesthetized by induction with acepromazine (0.01 mg/kg, subcutaneously), and surgical plane anesthesia was maintained with 1 to 5% isoflurane via endotracheal tube. Aseptic procedures were used. The dogs were placed in dorsal recumbency, and the ventral thoracic and abdominal regions were shaved and scrubbed with betadine solution before surgical draping of the surgical site. Following intubation, an intravenous catheter was placed for continuous delivery of 2 ml/kg per hour of lactated Ringer’s solution. Normal body temperature was maintained by use of warm water recirculating heating pads. Physiologic parameters were monitored and recorded at least every 15 min. Before the procedure, an orogastric tube was used to decompress the stomach. Perioperative antibiotic prophylaxis included 25 mg/kg of Cefazolin (intravenously). The esophagus was isolated through an upper midline laparotomy (extending from the umbilicus to the xiphoid process). The lower third of the esophagus was mobilized carefully to avoid injury to the vagus nerve. The right and left crus were dissected and transected to create an enlarged esophageal hiatus that naturally mimicked a type 1 sliding paraesophageal hiatal hernia. A gastropexy was placed to secure the body of the stomach to the abdominal wall 2 cm below the GEJ with nonabsorbable 2-0 sutures. The muscle fibers of the lower esophageal sphincter were cut (Heller myotomy). The muscular plane of the ventral abdominal wall was then closed with sutures placed in a continuous pattern with polyglactin or polypropylene 2-0 to 1 suture material. Grafts were used to close the skin (Covidien).

During the same surgical procedure, an endoscopy was performed to create a mucosal defect in the distal esophagus. An area of 2 cm circumferential by 3 cm longitudinal was identified 1 cm above the GEJ on the anterior side of the esophagus. Using a Duette band-ligation endoscopic mucosal resection (EMR) kit (Cook Medical), the mucosa was removed. The area was marked by submucosal injection of Spot Endoscopic Marker (GI Supply) at opposing corners. Following the surgical procedure, the animal was continually monitored for 24 hours, recording the physiological parameters every 3 hours. All animals received buprenorphine [0.01 mg/kg twice a day (BID)] and oral cephalexin (35 mg/kg for 5 days after the procedure. Weight after the initial surgery was recorded daily and three times a week thereafter.

After completing the buprenorphine and cephalexin, all animals received 100 μg of pentagastrin subcutaneously daily for at least 90 days until ECM hydrogel treatment was initiated or the equivalent time point for the control animals. After the surgery, dogs were monitored monthly to evaluate the progress of the reflux inducing procedure (fig. S1).

### Biopsy collection before treatment

Biopsies were collected before the start of treatment to determine the highest state of esophageal disease. Dogs were anesthetized by induction with acepromazine (0.01 mg/kg, subcutaneously), and surgical plane anesthesia was maintained with 1 to 5% isoflurane via endotracheal tube. Animals were placed with their neck extended so that the endoscope did not put undue pressure on the trachea or the nearby vessels. If necessary, insufflation with minimal amounts of air was done to achieve appropriate visualization. Biopsy samples were collected for all animals around the circumference of the esophagus at the same level by the EMR technique previously mentioned or with forceps. For biopsy retrieval, first the z line was identified to prevent collecting any stomach tissue. Under direct vision, biopsy forceps were advanced through the endoscope channel, opened, and withdrawn until the opened jaws were flush with the tip of the endoscope. The tip of the endoscope was flexed so that it was turned into the mucosa at as close to a 90° angle as possible. To draw the mucosa into the jaws of the forceps, the forceps were advanced into the mucosa until moderate resistance was felt. At this point, the jaws of the forceps were closed. The tip of the endoscope was straightened and withdrawn. The sample was retrieved from the endoscope. The location and circumferential position (anterior, posterior, etc.) of the biopsy and the time mark in the video were recorded for later identification and sample matching to location. All biopsy samples were fixed in formalin for >48 hours.

### eECM hydrogel treatment

Pentagastrin was discontinued in all animals and omeprazole therapy (20 mg daily) was initiated at day 0 (i.e., after each dog had received 90 days of pentagastrin treatment). Dogs received either eECM hydrogel (12 mg/ml and 25 ml, twice daily) orally at 15°C plus omeprazole or omeprazole only (i.e., control) for 30 days.

Treatment animals were administered ECM hydrogel per os. The eECM digest was thawed at 4°C, and the neutralizing solution was added, and the pre-gel was stirred at room temperature until it reached 15°C. At that point, a 60-ml catheter tip syringe was used to deliver the pre-gel to the dogs per os.

Dogs were restricted for food and water for the hour following administration of the hydrogel. The treatment schedule was designed to keep the dogs from eating immediately after the pre-gel administration. Animals were fed once in the morning and once in the afternoon, and the pre-gel was administered after the dogs had eaten to maximize exposure without food and without disturbing the animals’ normal feeding schedule.

### Safety

All animals were monitored daily for adverse gastroenterological events or complications throughout administration of the therapy. Weight was recorded, complete blood counts, and serum chemistry and electrolyte values (VETSCAN analysis) were monitored and recorded before and after treatment.

### Necropsy

Thirty days after starting treatment with eECM hydrogel and omeprazole or omeprazole alone, all animals underwent an endoscopy procedure to evaluate their esophagus before sacrifice. The procedure was recorded, and the anterior and posterior sides of the esophagus were identified. Blood was collected for analysis and the animal was euthanized with sodium pentobarbital (390 mg/kg).

After sacrifice, the esophagus was harvested, and directionality and in-body dimensions were maintained. The esophagus was split longitudinally. The anterior and posterior surfaces were identified on the opened esophagus and biopsy samples were collected using a 12-mm biopsy punch along the circumference of the esophagus (three samples per height) starting at the GEJ (including stomach tissue) and proximal area until the end of the defect ensuring all of the lower esophagus was adequately represented (figs. S1, B and C, and S5). The height from the GEJ and relation to the anterior side of all samples was recorded to allow identification of the location of the sample in the esophagus during subsequent analysis. All samples were fixed in neutral buffered formalin for at least 48 hours.

### Analysis of harvested tissue samples

Serial sections (5 μm) of biopsies (D0) and necropsy samples (D30) were stained with H&E and Alcian blue by standard histologic techniques and imaged with a bright-field microscope. A diagnosis of intestinal metaplasia requires the presence of goblet cells (*18*) and positive Alcian blue staining.

H&E-stained slides of all samples collected were assessed by a blinded pathologist, and their characteristics (e.g., metaplasia, dysplasia, gastric tissue, etc.) were recorded. After the samples had been evaluated, they were matched with the animal and esophageal location from which they had been harvested. This technique allowed for the matching of esophageal location of biopsy to the same esophageal location at the moment of necropsy without introduction of bias. The samples at areas of interest (i.e., Barrett’s metaplasia on the biopsy) were matched by location to the tissue at the time of necropsy and analyzed. These same samples were used for further immunolabeling analyses.

### Immunolabeling

Immunolabeling was performed using primary antibodies for squamous esophageal epithelial cell markers cytokeratin 13 (EPR3671) (rabbit monoclonal, Abcam, ab92551, 1:250) and cytokeratin 14 (LL002) (mouse monoclonal, Abcam, ab7800, 1:400) and the Barrett’s epithelial cell marker Sox9 (rabbit polyclonal, Chemicon, AB5535, 1:1000). Immunolabeling was also performed for TNFα (rabbit polyclonal, Novus Biologicals, NBP1-19532, 1:200) to identify the proinflammatory cell infiltrate. The secondary antibodies used were goat anti-rabbit 488 (Abcam, ab150077, 1:200) for CK13 and Sox9, donkey anti-mouse 488 (Invitrogen, A21202, 1:200) for CK14, and goat anti-rabbit 594 (Invitrogen, A11037, 1:200) for TNFα. All primary and secondary antibodies were diluted in blocking buffer solution (5% bovine serum albumin with 1% Tween 20/1% Triton X-100).

Serial sections (5 μm) of each tissue specimen identified previously were deparaffinized with xylene and rehydrated through a graded ethanol series. Antigen retrieval was performed using a citrate buffer [10 mM citric acid and 0.05% Tween 20 (pH 6)] brought to boil, and the slides were incubated for 20 min in the solution maintained at 95° to 100°C. The slides in the buffer solution were brought to room temperature and washed 3 × 5 min in 1 × PBS and blocked for 1 hour. The primary antibody solution was added and allowed to incubate overnight at 4°C. The slides were washed 3 × 5 min in PBS at room temperature, and the secondary antibody solution was added for 1 hour at room temperature. Slides were washed 5 × 5 min in PBS and counterstained with 4′,6-diamidino-2-phenylinidole (DAPI). Slides were washed 3 × 5 min in PBS, mounted in fluorescent mounting media (Dako, Glostrup, Denmark), and cover-slipped. The slides were imaged on a Nikon E600 fluorescence microscope with the Nuance FX Multispectral Imaging System for fluorescence. Spectral libraries were prepared for DAPI, Alexa Fluor 488, and Alexa Fluor 594 and unstained tissue autofluorescence. Images were spectrally unmixed using the prepared spectral libraries. Primary deletes were confirmed to be negatively stained.

Representative images were taken of the H&E, Alcian blue, CK13, CK14, and Sox9 staining at 10× and 20× fields. Three images per sample for TNFα were taken at 20× and quantified using a CellProfiler pipeline for positive TNFα and DAPI staining, with a threshold of 0.25 intensity or greater for the 594 channel to discount any background staining.

### Cytokine antibody array

Distal esophageal tissue and proximal normal tissue from each animal were flash-frozen at necropsy after 30 days of treatment and homogenized in cell lysis buffer, and soluble protein extract was probed with the Human Antibody Cytokine Array (Abcam, ab133997) for 42 targets according to the manufacturer’s instructions. Densitometry was performed on the microarray using Image Lab software (version 6.0.1, Bio-Rad Laboratories) to make semiquantitative comparisons. The array data were normalized to a reference array, and mean pixel intensity for each cytokine was presented as the relative expression (ratio) in the distal remodeled tissue compared to the animal’s own proximal, normal tissue for eECM hydrogel treatment (*n* = 6) and control (*n* = 2) with technical duplicates. Differentially expressed cytokines were defined as >2- or <−2-fold change.

### Statistical analysis

#### Rheology

A two-way ANOVA was performed for the main effects of ECM concentration and shear rate for the viscosity flow profile. A one-way ANOVA was performed for the main effect of ECM concentration on modulus for the time sweep test for maximum *G*′ and maximum *G*″. A one-way ANOVA was performed for the main effect of ECM concentration on gelation time. The post hoc Tukey’s multiple comparisons test was performed to determine the difference of the main effect for the one-way and two-way ANOVA. Data are presented as means ± SD (*n* = 3).

#### Mucoadhesive strength

A one-way ANOVA was performed for the main effect of ECM concentration, and the post hoc Tukey’s multiple comparisons test was performed to determine the difference of the main effect. Data are presented as means ± SD (*n* = 3).

#### Mucoadhesion with laminar flow

A two-way ANOVA was performed for the main effects of ECM concentration and time. The post hoc Tukey’s multiple comparisons test was performed comparing gel thickness after laminar flow of each concentration within each time. Data are presented as means ± SEM (*n* = 3), with six measurements per biological sample.

#### Immunolabeling

Student’s unpaired *t* test was performed comparing treatment animal specimens (*n* = 6) to control animal (*n* = 2) specimens. Data are presented as means ± SEM, with three images per biological sample.

#### Targeted proteomics

A two-way ANOVA was performed on protein expression for the independent variables sample type and functional classification or protein type, with post hoc multiple comparisons test. Data are presented as means ± SEM. Principal components analysis was performed by MetaboAnalyst (v4.0) (*60*).

## Supplementary Material

aba4526_Table_S2.xlsx

aba4526_Table_S1.xlsx

aba4526_SM.pdf

## SUPPLEMENTARY MATERIALS

Supplementary material for this article is available at http://advances.sciencemag.org/cgi/content/full/6/27/eaba4526/DC1

## Appendix

View/request a protocol for this paper from *Bio-protocol*.
