# Supplementary material for: Esophageal extracellular matrix hydrogel mitigates metaplastic change in a dog model of Barrett’s esophagus
Source: Sci Adv. 2020 Jul 1;6(27):eaba4526. doi: 10.1126/sciadv.aba4526 (PMC7329334; doi:10.1126/sciadv.aba4526)
Supplement: aba4526_SM.pdf [file aba4526_SM.pdf]

Science Advances
AAAS

advances.sciencemag.org/cgi/content/full/6/27/eaba4526/DC1

Supplementary Materials for

Esophageal extracellular matrix hydrogel mitigates metaplastic change in a dog model of Barrett's esophagus

Juan Diego Naranjo, Lindsey T. Saldin, Eric Sobieski, Lina M. Quijano, Ryan C. Hill, Patrick G. Chan, Crisanto Torres, Jenna L. Dziki, Madeline C. Cramer, Yoojin C. Lee, Rohit Das, Anant K. Bajwa, Rania Nossair, Molly Klimak, Lucile Marchal, Shil Patel, Sachin S. Velankar, Kirk C. Hansen, Kevin McGrath, Stephen F. Badylak*

*Corresponding author. Email: badylaks@upmc.edu

Published 1 July 2020, *Sci. Adv.* **6**, eaba4526 (2020)
DOI: 10.1126/sciadv.aba4526

The PDF file includes:

Figs. S1 to S6
Legends for tables S1 and S2

Other Supplementary Material for this manuscript includes the following:

(available at advances.sciencemag.org/cgi/content/full/6/27/eaba4526/DC1)

Tables S1 and S2

Supplementary Materials:

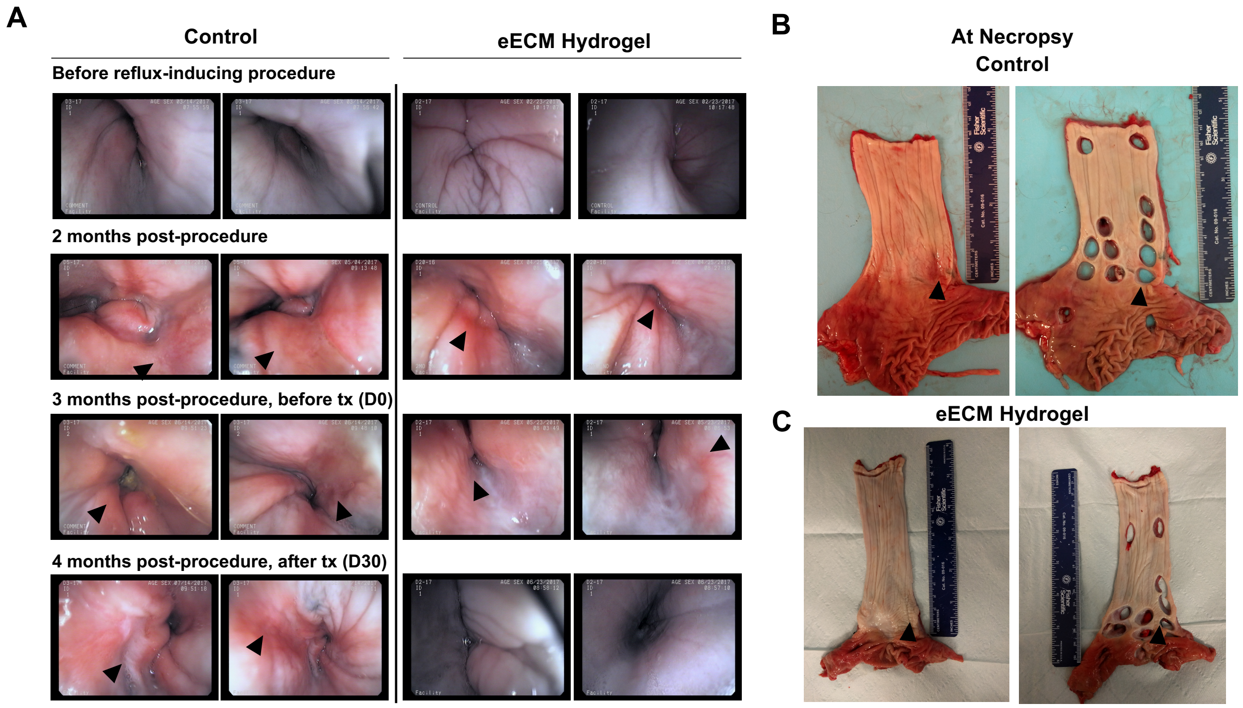

Fig. S1. Overview of injury progression throughout study for control and treatment animals. (A) Injury model created macroscopic esophagitis with ulcerations in the lower esophagus on both control and treated animals (up to 3 months). Ulceration remained visible in the control animal after 4 months macroscopically compared to normal esophageal tissue in the eECM hydrogel treated animal at the end of the study. The esophagus was harvested from the animals and opened longitudinally. The entire lower circumference of the esophagus was collected for analysis using 12mm biopsy punches, ensuring that the whole circumference was evaluated. **(B)** Macroscopic evaluation confirmed minor ulceration in the control animal (arrow) and **(C)** normal tissue in the eECM treated animal (arrow). (Photo credit: Juan Diego Naranjo, University of Pittsburgh).

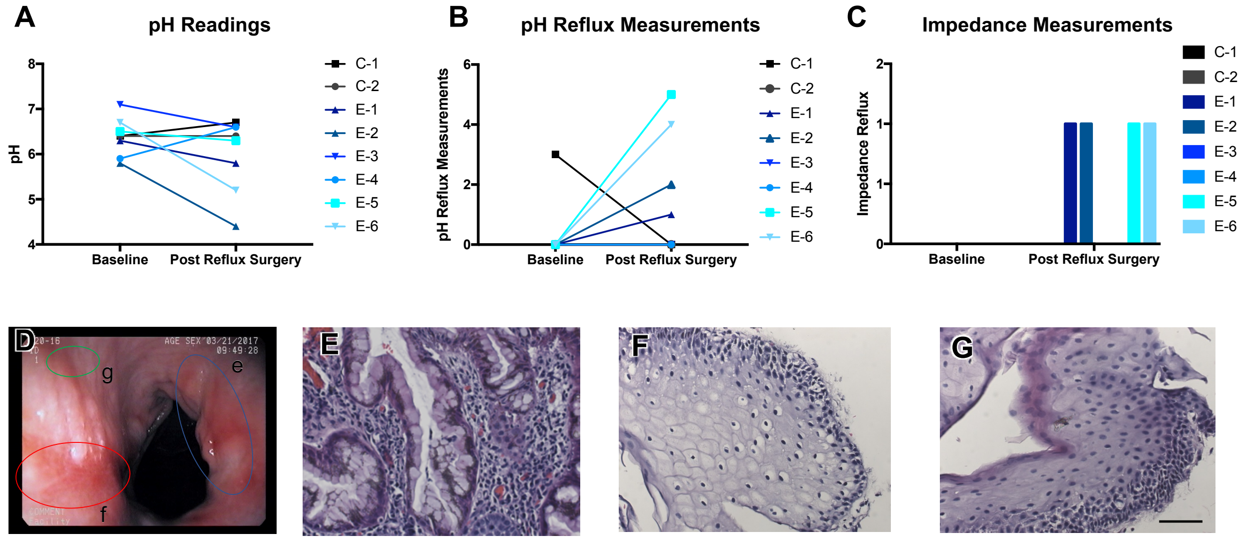

Fig. S2. Validation of the Barrett's esophagus canine model. Induction of reflux and columnar metaplasia was confirmed in the canine model of Barrett's esophagus**. (A)** One-hour pH readings were obtained for all animals before ("baseline") and after reflux-inducing surgery. Average pH before the procedure was 6.4±0.41 at baseline and 6.2± 0.57 post reflux-inducing surgery. Data are means ± SD (n=8), with absolute values for each animal shown in the graphs. All animals except E-4 and C-1 had a decrease in pH post reflux-inducing surgery when compared to baseline. **(B)** Reflux events were measured with the pH probe for 1h. One animal (C-1) showed a reflux event pre-operatively at baseline, and 4 animals (E-1, E-2, E-5, and E-6) presented at least one reflux episode as measured by pH. **(C)** No animals had any impedance events at baseline during the 1-hour readings. Four of 8 dogs presented at least one reflux episode as measured by impedance post reflux-inducing surgery (E-1, E-2, E-5, E-6). **(D)** A representative image of the distal esophagus at endoscopy before treatment. Biopsies were taken at an area of (*e*) esophagitis, (*f*) defect and (*g*) normal. (Photo credit: Juan Diego Naranjo, University of Pittsburgh). **(E)** H&E

stained biopsy harvested at area of esophagitis. **(F)** H&E stained biopsy harvested at site of defect. **(G)** Normal tissue stained with H&E. Scale bar= 50 μm.

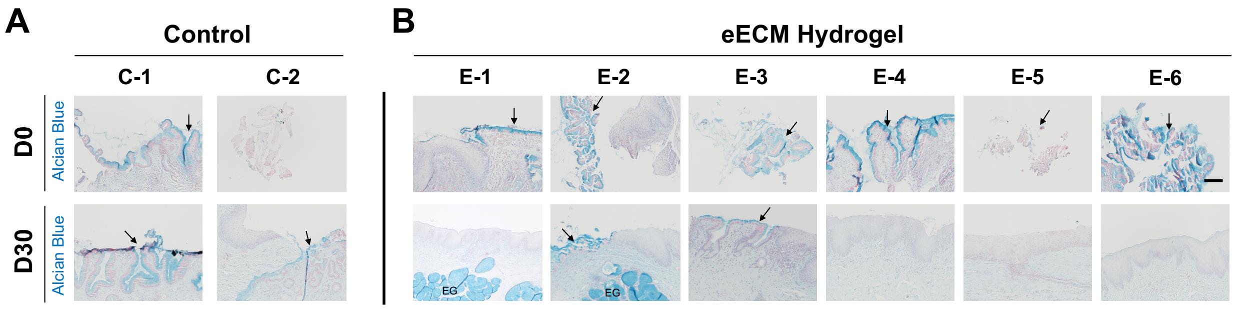

Fig. S3. Alcian blue staining. Biopsies were taken before treatment (D0), and location-matched samples were collected after 30 days of treatment (D30) for **(A)** control animals and **(B)** eECM hydrogel treated animals. Samples were stained with alcian blue, which labels the intracytoplasmic acidic mucin of goblet cells. Arrows indicate goblet cells characteristic of intestinal metaplasia and Barrett's esophagus. Non-metaplastic, mucin containing esophageal glands (EG) can also stain positively for alcian blue and are not a marker of Barrett's esophagus. Scale bar = 100 μm.

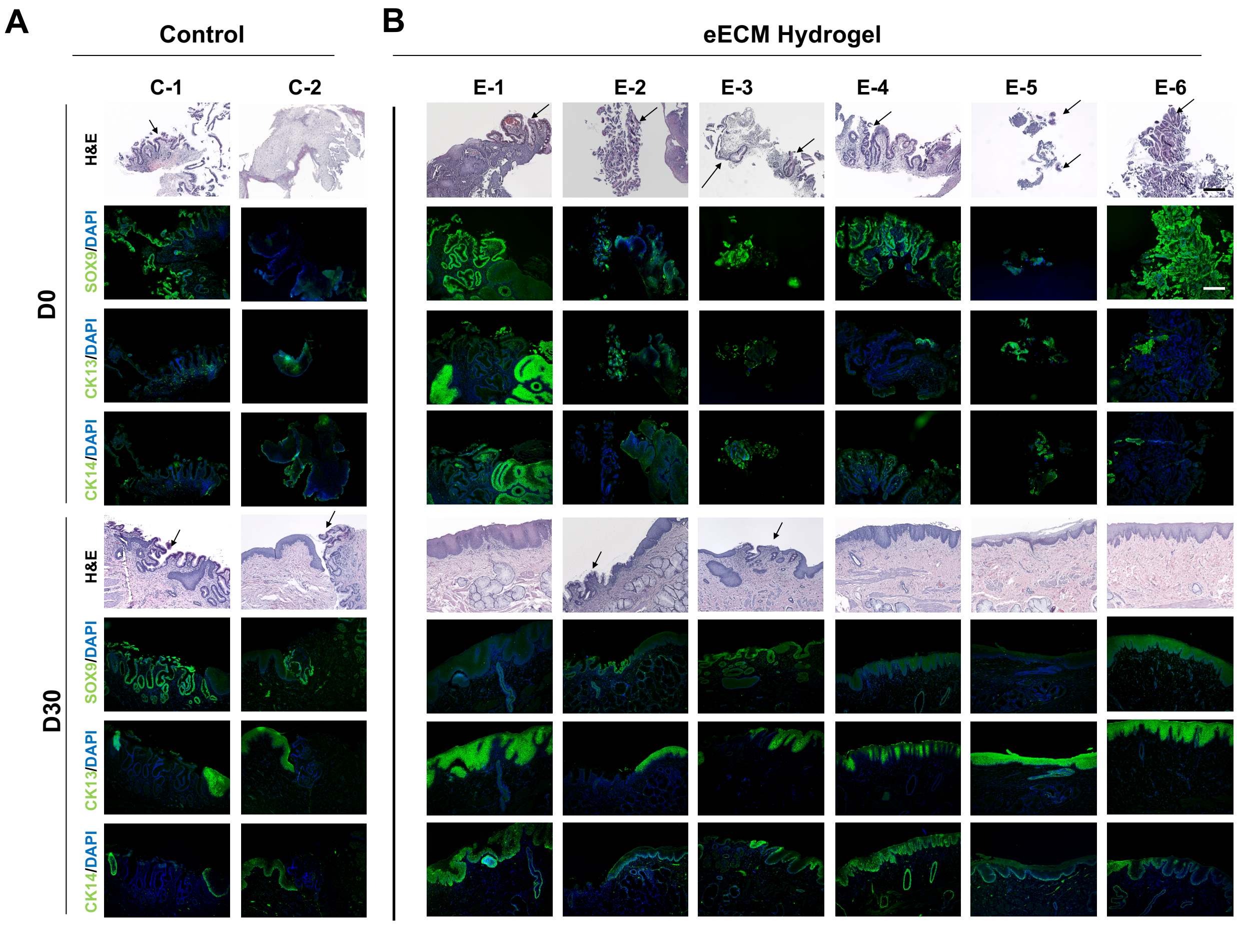

Fig. S4. Effect of eECM hydrogel treatment on esophageal epithelial cell phenotype after 30 days. Biopsies were taken before treatment (D0), and location-matched samples were collected after 30 days of treatment (D30) for **(A)** control animals and **(B)** eECM hydrogel treated animals. Samples were stained with hematoxylin and eosin (H&E), Barrett's marker Sox9, or normal esophageal squamous epithelial markers CK13 and CK14. Arrows indicate goblet cells characteristic of intestinal metaplasia and Barrett's esophagus. Scale bar = 250 µm.

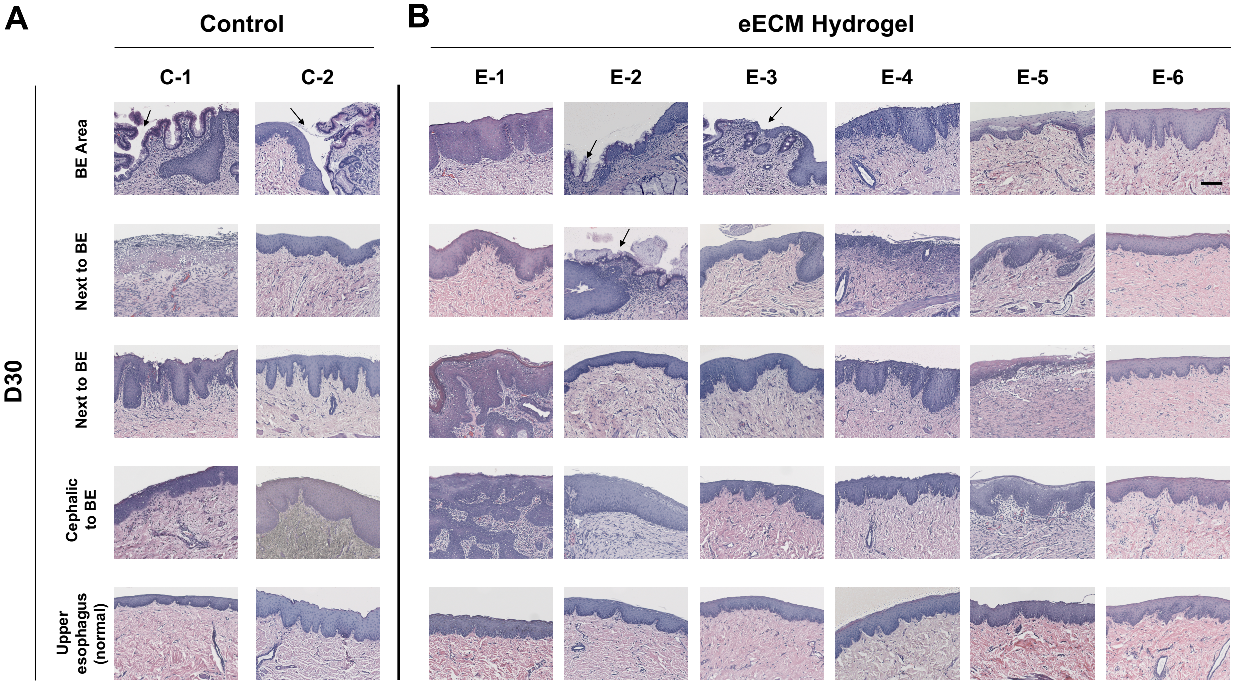

Fig. S5. Histologic view of the circumference of the distal esophagus. The entire circumference of the esophagus was collected after 30 days of treatment (D30) for **(A)** control and **(B)** eECM treated animals. Columnar cells were present in the main BE area in C-1, C-2, E-2 and E-3 where a transitional epithelium was seen. The only animal that had a columnar epithelium expanding from the BE area was E-2. Scale bar = 100 μm.

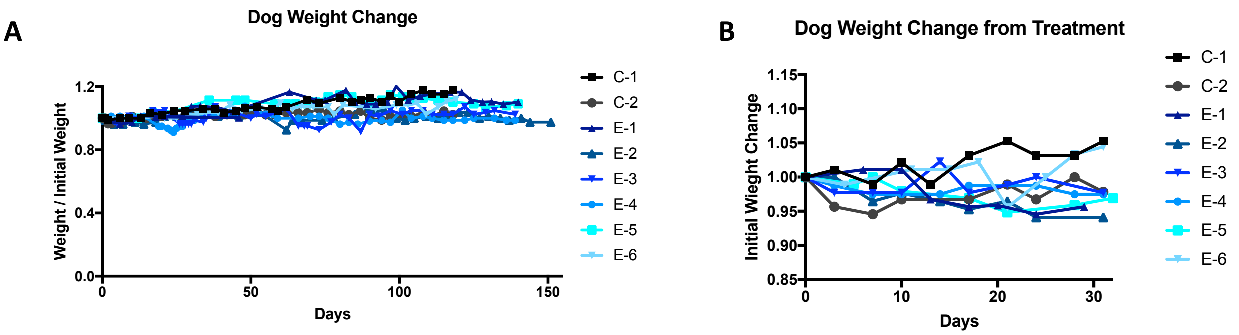

C

ANALYSIS	Before ECM Treatment (n=3)	After 30 days ECM Treatment (n=3)	Normal Range	Units
ALB (Albumin)	3.325	3.4	2.5 - 4.4	G/DL
ALP (Alkaline Phosphatase)	15	17.33	20 – 150	U/L
ALT (Alanine aminotransferase)	42.25	57.67	10 – 118	U/L
AMY (Amylase)	339	427	200 – 1200	U/L
TBIL (Total Bilirubin)	0.275	0.2	0.1 – 0.6	MG/DL
BUN (Blood urea nitrogen)	12.75	11.67	7 – 25	MG/DL
CA (Calcium)	10.15	10.2	8.6 – 11.8	MG/DL
PHOS (Phosphorus)	4.9	4.17	2.9 – 6.6	MG/DL
CRE (Creatinine)	0.75	0.87	0.3 – 1.4	MG/DL
GLU (Glucose)	92.75	112	60 – 110	MG/DL
NA^+ (Sodium)	153	137. 67	138 – 160	MMOL/L
K+ (Potassium)	4.35	4	3.7 – 5.8	MMOL/L
GLOB (globulin)	1.525	1.7	2.3 – 5.2	G/DL

Fig. S6. Safety of 30 days of eECM administration. **(A)** Weight recording for all animals during the period of study. There was no overall weight change with a final weight of 106.2% ±6.6%. **(B)** Animal weights during eECM administration. There was no change with a 98.7%±3.8% after completing treatment. **(C)** A veterinary blood scan panel did not show any physiological parameters out of range after treatment (n=3).

Protein	GENE	Uniprot	Gene Ontology Class	Matrisome	Protein Abundance (nmol/g)*						Trend	Tech CV (%)
					Native			eECM				
					n1	n2	n3	n1	n2	n3		
Agrin(iso 2,3,4,5,&6)	AGRN^	O00468	Basement Membrane	Glycoprotein	1.71	1.73	1.96	0.34	0.57	0.45		31%
Collagen alpha-1/5(IV) chain(Arresten/Core Protein)	COL4A1/5^	160613	Basement Membrane	Collagen	2.62	3.13	3.73	0.36	0.27	0.07		6%
Collagen alpha-1(IV) chain(Arresten/Core Protein)	COL4A1^	160613	Basement Membrane	Collagen	0.37	0.42	0.32	0.80	0.36	0.45		8%
Collagen alpha-2(IV) chain	COL4A2	P08122	Basement Membrane	Collagen	0.52	0.63	0.61	0.91	0.20	0.22		10%
Collagen alpha-2(IV) chain(Canstatin/Core Protein)	COL4A2^	161386	Basement Membrane	Collagen	0.73	1.11	0.80	1.59	0.70	0.64		4%
Perlecan	HSPG2	375271	Basement Membrane	Proteoglycan	3.23	2.43	2.41	0.36	0.08	0.07		9%
Laminin alpha-2	LAMA2	P24043	Basement Membrane	Glycoprotein	0.04	0.04	0.04					17%
Laminin alpha-3	LAMA3	Q61789	Basement Membrane	Glycoprotein	0.07	1.01		0.30	0.57	0.16		13%
Laminin alpha-4	LAMA4	201819	Basement Membrane	Glycoprotein	0.18	0.23	0.85			0.23		6%
Laminin alpha-5	LAMA5	O15230	Basement Membrane	Glycoprotein	0.07	0.23	0.08	0.07				19%
Laminin Beta-1	LAMB1	197090	Basement Membrane	Glycoprotein	0.50	1.31	0.47	0.22	0.35	0.27		13%
Laminin Beta-2	LAMB2	196474	Basement Membrane	Glycoprotein	0.79	0.93	0.70	0.03	0.03			13%
Laminin Gamma-1	LAMC1	177387	Basement Membrane	Glycoprotein	0.84	1.17	0.97	0.73	0.28	0.24		8%
Laminin Gamma-2	LAMC2	Q13753	Basement Membrane	Glycoprotein	2.01	3.48	3.58		1.30	0.37		11%
Nidogen-1	NID1	137039	Basement Membrane	Glycoprotein	0.26	0.31	0.24	0.08	0.03			16%
Nidogen-2	NID2	Q14112	Basement Membrane	ECM Glycoproteins	1.88	2.19	1.73					8%
Collagen alpha-1(XII) chain	COL12A1	340214	FACIT Collagen	Collagen	0.59	0.51	0.61	0.30	0.66	0.52		15%
Collagen alpha-1(XIV) chain	COL14A1	191772	FACIT Collagen	Collagen	7.71	8.96	8.70					12%
Collagen alpha-1(I) chain	COL1A1	137953	Fibrillar Collagen	Collagen	237.83	232.07	252.01	292.35	327.58	368.50		4%
Collagen alpha-1(I) chain(GalGlu-K)	COL1A1^	137953	Fibrillar Collagen	Collagen	0.20	0.18	0.12					11%
Collagen alpha-2(I) chain	COL1A2	129564	Fibrillar Collagen	Collagen	152.85	132.47	160.71	188.21	220.13	237.05		4%
Collagen alpha-2(I) chain(C-term Propeptide)	COL1A2^	129564	Fibrillar Collagen	Collagen	2.11	1.76	1.63					24%
Collagen alpha-1(III) chain	COL3A1	138936	Fibrillar Collagen	Collagen	122.05	113.94	72.61	115.35	142.76	158.29		8%
Collagen alpha-1(V) chain	COL5A1	183987	Fibrillar Collagen	Collagen	3.94	4.06	3.84	8.81	7.50	10.45		2%
Collagen alpha-2(V) chain	COL5A2	145018	Fibrillar Collagen	Collagen	1.86	1.70	1.54	3.98	3.97	4.42		7%
Fibulin 5	FBLN5	50160	Matricellular	Glycoprotein	0.48	0.28	0.29					25%
Periostin	POSTN	93155	Matricellular	Glycoprotein	1.03	0.90	0.29	0.99	0.90	0.48		16%
Secreted protein, acidic, cysteine-rich (osteonectin)	SPARC	34296	Matricellular	Glycoprotein	0.05	0.08	0.08					9%
Thrombospondin 2	THBS2	P35442	Matricellular	Glycoprotein			0.04					12%
Tenascin-X	TNXB	126729	Matricellular	Glycoprotein	0.12	0.15	0.09					13%
Annexin A2	ANXA2	38678	Other ECM	ECM-affiliated	6.24	6.06	6.58	0.40	0.29	0.16		17%
Asporin	ASPN	42573	Other ECM	Proteoglycan	1.20	1.24	1.03					16%
Galectin-1	LGALS1	14857	Other ECM	ECM-affiliated	9.54	16.52	7.98					10%
Galectin-3	LGALS3	27202	Other ECM	ECM-affiliated	6.76	7.07	7.15					29%
Mimecan/Osteoglycin	OGN	34069	Other ECM	Proteoglycan	9.00	10.96	9.86					8%
Biglycan	BGN	41706	Microfibril-Associated	Proteoglycan	0.92	1.44	0.91					20%
Collagen alpha-1(XVIII) chain	COL18A1	182881	Microfibril-Associated	Collagen	0.05	0.06	0.04					6%
Collagen alpha-1(VI) chain	COL6A1	108806	Microfibril-Associated	Collagen	15.81	22.33	17.56	3.90	1.50	1.46		9%
Collagen alpha-2(VI) chain	COL6A2	108579	Microfibril-Associated	Collagen	14.57	15.98	15.30	0.83	0.35	0.16		7%
Collagen alpha-3(VI) chain	COL6A3	288133	Microfibril-Associated	Collagen	16.49	21.23	17.03	18.48	10.28	8.16		3%
Collagen alpha-1(VIII) chain	COL8A1	P27658	Microfibril-Associated	Collagens	0.94	1.36	0.53	0.63	0.57	0.37		15%
Decorin	DCN	39805	Microfibril-Associated	Proteoglycan	5.57	3.97	3.45					5%
Dermatopontin	DPT	24203	Microfibril-Associated	Glycoprotein	3.50	3.21	3.30	4.62	3.03	3.65		10%
Emilin 1	EMILIN1	106667	Microfibril-Associated	Glycoprotein	0.05	0.07	0.03					7%
Fibrillin 1	FBN1	311952	Microfibril-Associated	Glycoprotein	0.63	0.48	0.50	0.67	0.58	0.87		6%
Fibromodulin	FMOD	Q06828	Microfibril-Associated	Proteoglycans	9.30	6.95	5.41					12%
Fibronectin 1(Anastellin, type-III 4/7/9/13)	FN1	P02751	Microfibril-Associated	Glycoprotein	1.38	2.65	1.39	3.65	1.91	1.27		9%
Latent transforming growth factor beta binding protein 1	LTBP1	Q14766	Microfibril-Associated	Glycoprotein	0.14	0.03	0.05	0.04		0.14		9%
Lumican	LUM	38279	Microfibril-Associated	Proteoglycan	9.83	12.86	12.74	0.03		0.16		7%
Microfibrillar-associated protein 2	MFAP2	20578	Microfibril-Associated	Glycoprotein	2.05	1.89	1.96					14%
Prolargin	PRELP	43179	Microfibril-Associated	Proteoglycan	7.62	5.74	6.70	0.10	0.04	0.06		5%
Versican	VCAN	300008	Microfibril-Associated	Proteoglycans	0.37	0.52	0.30					13%
Target of Nesh-SH3	ABI3BP	Q7Z7G0	Secreted	ECM Glycoproteins	1.06	1.17	0.78					29%
Transforming growth factor-beta-induced protein ig-h3	TGFBI	74597	Secreted	Glycoprotein	0.53	0.86	0.13	0.28		0.05		12%
Actin (All Isoforms)	ACT	42051	Cytoskeletal	Cellular	146.62	253.09	147.65	0.32	0.10	0.11		4%
Actin, cytoplasmic 1/2	ACTB	41737	Cytoskeletal	Cellular	32.82	60.80	33.55	1.14	0.53	0.21		12%
Myosin(Myosin-3,4,6,7)	MYH^	P12882	Cytoskeletal	Cellular	6.54	1.43	1.10					10%
Spectrin alpha chain, non-erythrocytic 1	SPTA2	284637	Cytoskeletal	Cellular	1.22	1.46	1.27	0.42	0.50	0.33		6%
Tubulin beta-4B chain(4b & 5 chain)	TUBB^	49586	Cytoskeletal	Cellular	1.97	2.80	2.09	0.14	0.08	0.04		2%
Vimentin	VIM	53733	Cytoskeletal	Cellular	3.51	3.68	3.35	0.04	0.08	0.07		6%
Glyceraldehyde-3-phosphate dehydrogenase	GAPDH	35828	Other Cellular	Cellular	67.15	72.07	43.30					6%
Histone H1(H1.1,H1.2,H1.3,H1.4)	H1^	20863	Other Cellular	Cellular	49.67	58.69	31.24					7%
Histone 2A(H2A-A-K)	H2A^	14077	Other Cellular	Cellular	15.39	15.42	9.97					7%
Lamin-A/C	LMNA		Other Cellular	Cellular	50.40	12.28	16.02	0.54		0.05		16%

^indicates peptide that was used to identify protein may be homologous to multiple protein isoforms, cryptic peptides, or susceptiple to PTM and is explained in paranthesis next to the protein name

* per gram of dry weight

Table S1. Quantitative proteomics of porcine eECM and esophageal mucosal tissue.

Absolute quantification of proteins for native esophageal mucosa tissue and eECM (n=3), shown normalized to dry weight. Proteins are separated by gene ontology class and labeled by their matrisome classification. Red represents highly expressed native esophageal mucosa proteins, blue represents highly expressed eECM proteins, and graphed under "Trend." Tech CV refers to coefficient of variance.

Protein	GENE	Uniprot	Gene Ontology Class	Matrisome	Protein Abundance as Ratio Distal/Normal						
					C-1	E-1	E-2	E-3	E-4	E-5	E-6
Collagen alpha-1(IV) chain(Arresten/Core Protein)	COL4A1*	P02462	Basement Membrane	Collagen	1.30	1.19	1.81	2.08	1.30	1.10	1.43
Perlecan(Endorepellin)	HSPG2*	P98160	Basement Membrane	Proteoglycan	1.83	1.73	2.24	2.35	1.23	1.54	1.50
Nidogen 1/2 (osteonidogen)(Nid1/2)	NID1/2*	Q14112	Basement Membrane	Glycoprotein	1.43	2.05	2.06	2.50	1.26	1.45	2.09
Collagen alpha-1/5(IV) chain(Arresten/Core Protein)	COL4A1/5*	P02462	Basement Membrane	Collagen	0.85	2.01	1.51	1.89	1.51	1.39	1.37
Collagen alpha-1/5(IV) chain(Arresten/Core Protein)	COL4A1/5*	P02462	Basement Membrane	Collagen	0.84	1.71	1.42	1.71	1.54	1.27	1.41
Collagen alpha-2(IV) chain(Canstatin/Core Protein)	COL4A2*	P08572	Basement Membrane	Collagen	1.40	1.16	1.66	2.46	1.32	1.12	1.54
Agrin	AGRN	O00468	Basement Membrane	Glycoprotein	2.35	0.98	1.55	1.47	1.01	1.25	1.70
Collagen alpha-5(IV) chain	COL4A5	P29400	Basement Membrane	Collagen	0.38	4.67	2.92	2.98	0.88	1.57	1.50
Perlecan	HSPG2	P98160	Basement Membrane	Proteoglycan	1.67	1.73	2.36	2.36	1.31	1.67	1.38
Laminin alpha-4	LAMA4	Q16363	Basement Membrane	Glycoprotein	1.16	1.98	1.49	2.01	1.21	1.22	2.27
Laminin Beta-1	LAMB1	P07942	Basement Membrane	Glycoprotein	1.67	2.11	1.11	0.91	1.10	1.03	2.00
Laminin Beta-2	LAMB2	Q61292	Basement Membrane	Glycoprotein	0.84	2.88	1.77	1.91	1.23	1.32	1.63
Laminin Gamma-1	LAMC1	P11047	Basement Membrane	Glycoprotein	1.43	1.61	1.63	2.26	1.12	1.27	1.69
Nidogen-1	NID1	P14543	Basement Membrane	Glycoprotein	0.88	1.61	2.01	3.07	1.11	1.02	3.01
Myosin(Myosin-3,4,6,7)	MYH*	P12882	Cytoskeletal	Cellular	eECM Only	Normal Only	eECM Only				
Tubulin beta-4B chain(4b & 5 chain)	TUBB*	P04350	Cytoskeletal	Cellular	1.72	0.69	1.40	1.66	1.13	1.12	1.86
Actin (All Isoforms)	ACT	P60709	Cytoskeletal	Cellular	1.14	1.17	1.68	2.13	1.13	1.10	2.05
Actin, cytoplasmic 1/2	ACTB	P60709	Cytoskeletal	Cellular	2.53	0.86	1.66	2.08	1.03	1.30	2.60
Desmin	DES	P17661	Cytoskeletal	Cellular	0.95	0.80	1.06	0.97	0.96	0.98	1.04
Spectrin alpha chain, non-erythrocytic 1	SPTA2	Q13813	Cytoskeletal	Cellular	1.41	0.90	1.73	1.52	1.18	1.36	1.30
Vimentin	VIM	P08670	Cytoskeletal	Cellular	3.07	0.54	2.12	2.43	1.03	1.73	2.11
Lysyl oxidase-like 1	LOXL1	Q08397	ECM regulator	ECM regulator	2.59	1.52	3.91	1.59	1.02	2.12	1.70
Transglutaminase 2	TGM2	P21980	ECM regulator	ECM regulator		Normal Only					
Annexin A2	ANXA2	P07355	ECM-affiliated	ECM-affiliated	1.09	0.68	1.11	1.39	0.99	0.85	2.17
Asporin	ASPN	Q9BXN1	ECM-affiliated	Proteoglycan	3.09	0.82	1.88	1.85	0.84	1.96	1.94
Extracellular Matrix Protein 1	ECM1	Q62894	ECM-affiliated	Glycoprotein	0.94	0.87	2.10	1.24	1.30	1.00	2.88
Galectin-1	LGALS1	P09382	ECM-affiliated	ECM-affiliated	2.62	0.99	1.84	2.06	0.98	1.44	2.01
Mimecan/Osteoglycin	OGN	P20774	ECM-affiliated	Proteoglycan	0.57	0.98	0.94	1.13	0.90	0.79	1.93
Collagen alpha-1(XII) chain	COL12A1	Q99715	FACIT Collagen	Collagen	7.26	0.19	2.12	1.65	0.73	1.30	1.25
Collagen alpha-1(XIV) chain	COL14A1	Q05707	FACIT Collagen	Collagen	3.06	1.12	4.32	3.80	0.73	3.77	2.83
Collagen alpha-2(I) chain(C-term Propeptide)	COL1A2*	P08123	Fibrillar Collagen	Collagen	6.14	0.92	3.25	8.78	1.00	5.78	1.54
Collagen alpha-1(I) chain(C-term Propeptides (NC1 Domai	COL1A1*	P02452	Fibrillar Collagen	Collagen	5.03	0.76	2.97	6.42	0.84	6.59	1.36
Collagen alpha-2(I) chain(C-term Propeptide)	COL1A2*	P08123	Fibrillar Collagen	Collagen	8.76	0.69	4.61	11.70	1.30	6.86	1.58
Collagen alpha-1(I) chain	COL1A1	P02452	Fibrillar Collagen	Collagen	0.76	0.77	0.76	1.11	1.03	0.93	0.87
Collagen alpha-2(I) chain	COL1A2	P08123	Fibrillar Collagen	Collagen	0.83	0.74	0.80	1.14	0.93	1.01	0.98
Collagen alpha-1(V) chain	COL5A1	P20908	Fibrillar Collagen	Collagen	1.08	0.83	0.95	1.14	1.00	1.31	0.87
Collagen alpha-2(V) chain	COL5A2	P05997	Fibrillar Collagen	Collagen	1.26	0.82	0.98	1.35	1.08	1.14	1.00
Collagen alpha-1(III) chain	COL3A1	P02461	Fibrillar Collagen	Collagen	0.91	0.79	0.93	1.26	0.89	1.35	0.78
Tenascin-C(Iso1,2,3,4,5)	TNC*	P24821	Matricellular	Glycoprotein	4.51	0.23	1.17	1.07	1.19	0.67	3.79
Fibulin 5	FBLN5	Q9WVH8	Matricellular	Glycoprotein	4.23	0.68	2.92	3.11	0.69	1.80	2.83
Periostin	POSTN	Q62009	Matricellular	Glycoprotein	4.86	0.33	2.19	2.25	0.87	1.44	2.48
Secreted protein, acidic, cysteine-rich (osteonectin)	SPARC	P09486	Matricellular	Glycoprotein		Normal Only					
Thrombospondin 1	THBS1	P07996	Matricellular	Glycoprotein	1.14	0.66	0.30	0.91	1.17	0.50	1.77
Thrombospondin 2	THBS2	P35442	Matricellular	Glycoprotein	eECM Only		eECM Only				
Tenascin-X	TNXB	P22105	Matricellular	Glycoprotein	1.66	0.61	0.65	1.18	0.90	0.73	2.59
Histone 2A(H2A-A-K)	H2A*	Q96QV6	Other Cellular	Cellular	2.59	0.59	1.54	1.68	1.06	1.28	1.83
Histone H1(H1.1,H1.2,H1.3,H1.4)	H1*	P16403	Other Cellular	Cellular	2.01	0.52	1.42	1.33	1.35	0.61	1.36
Alpha/gamma-enolase	ENO1/2	P09104	Other Cellular	Cellular		Normal Only					
Glyceraldehyde-3-phosphate dehydrogenase	GAPDH	P04406	Other Cellular	Cellular	0.64	0.48	1.07	1.71	0.56	1.40	0.59
Transforming growth factor-beta-induced protein ig-h3	TGFBI	Q15582	Secreted	Glycoprotein	1.95	0.63	1.00	1.18	0.76	0.66	3.21
Collagen alpha-1(VII) chain(Fibronectin type-III 3 Domain)	COL7A1*	Q63870	Microfibril-Associated	Collagen	2.28	0.38	Normal Only	2.38	1.23	0.49	1.55
Fibronectin 1(type-III 3/4/7//9/13 domain)	FN1	P02751	Microfibril-Associated	Glycoprotein	3.91	0.84	2.14	3.12	1.05	1.76	2.10
Latent transforming growth factor beta binding protein 1	LTBP1*	Q14766	Microfibril-Associated	Glycoprotein	eECM Only		eECM Only	eECM Only			
Biglycan	BGN	P21810	Microfibril-Associated	Proteoglycan	4.18	0.58	1.82	2.46	1.25	1.40	1.41
Chondroadherin	CHAD	O15335	Microfibril-Associated	Proteoglycan		eECM Only					
Collagen alpha-1(XVII) chain	COL17A1	Q9UMD9	Microfibril-Associated	Collagen	0.69	0.32	0.19	1.04	0.91	0.53	1.27
Collagen alpha-1(XVIII) chain	COL18A1	P39060	Microfibril-Associated	Collagen	2.22	1.03	1.78	1.62	0.99	1.34	1.32
Collagen alpha-1(VI) chain	COL6A1	P12109	Microfibril-Associated	Collagen	2.37	0.89	1.28	1.79	1.16	1.37	2.39
Collagen alpha-2(VI) chain	COL6A2	P12110	Microfibril-Associated	Collagen	2.17	0.68	1.38	2.52	1.05	1.28	2.13
Collagen alpha-3(VI) chain	COL6A3	P12111	Microfibril-Associated	Collagen	2.36	0.88	1.19	1.65	1.04	1.31	1.97
Decorin	DCN	Q01129	Microfibril-Associated	Proteoglycan	0.59	0.69	0.61	1.18	1.01	0.85	1.37
Dermatopontin	DPT	Q07507	Microfibril-Associated	Glycoprotein	0.88	0.91	0.77	1.41	0.89	0.95	1.14
Fibulin 3	EFEMP1	Q12805	Microfibril-Associated	Glycoprotein	2.99	0.81	3.43	1.80	0.73	1.51	2.70
Fibulin 4	EFEMP2	Q5XI84	Microfibril-Associated	Glycoprotein	2.07	0.90	3.10	1.54	0.98	1.17	1.60
Emilin 1	EMILIN1	Q9Y6C2	Microfibril-Associated	Glycoprotein	2.21	1.12	2.31	1.70	0.88	1.18	1.85
Fibulin 1	FBLN1	P23142	Microfibril-Associated	Glycoprotein	2.20	0.94	2.45	2.00	0.93	1.60	2.40
Fibrillin 1	FBN1	Q61554	Microfibril-Associated	Glycoprotein		Normal Only					
Fibromodulin	FMOD	Q06828	Microfibril-Associated	Proteoglycan	0.44	0.65	0.59	1.65	0.65	1.28	1.17
Fibronectin 1	FN1	P02751	Microfibril-Associated	Glycoprotein	0.33	1.19	0.31	7.05	0.86	1.19	2.72
Lumican	LUM	P51885	Microfibril-Associated	Proteoglycan	1.02	0.84	1.00	1.27	1.07	0.95	1.36
Microfibrillar-associated protein 2	MFAP2	P55001	Microfibril-Associated	Glycoprotein	3.33	0.88	1.69	1.98	0.94	1.66	1.85
Prolargin	PRELP	Q9JK53	Microfibril-Associated	Proteoglycan	0.37	0.94	0.85	1.22	1.02	0.93	1.40
Osteopontin	SPP1	P10923	Microfibril-Associated	Glycoprotein	0.98	0.04	1.01	0.99	0.92	0.97	1.00
Versican	VCAN	P13611	Microfibril-Associated	Proteoglycans	2.80	0.97	1.75	4.64	1.28	2.29	3.71
Vitronectin	VTN	Q3ZBS7	Microfibril-Associated	Glycoprotein	1.93	1.29	1.39	1.55	1.09	1.67	1.50

Table S2. Quantitative proteomics of canine distal and normal tissue. Absolute quantification of proteins (nmol/g), shown as a ratio of distal/normal within each dog for eECM treatment animals (n=6) and control (n=1). Proteins are separated by gene ontology class and

labeled by their matrisome classification. Red represents highly expressed proteins in the distal remodeled tissue, and blue represents highly expressed proteins in the normal tissue.
